# Transcriptome analysis reveals salivary gland-specific neuropeptide signaling genes in the predatory stink bug, *Picromerus lewisi*

**DOI:** 10.3389/fphys.2023.1270751

**Published:** 2023-09-29

**Authors:** Wenhong Li, Zhimo Li, Xiang Yang, Xinyi Wang, Mingwei Yang, Chunyang Huang, Yueping He

**Affiliations:** ^1^ Institute of Plant Protection, Guizhou Academy of Agricultural Sciences, Guiyang, China; ^2^ Guizhou Provincial Tobacco Company Zunyi Branch, Zunyi, China; ^3^ Hubei Insect Resources Utilization and Sustainable Pest Management Key Laboratory, College of Plant Science and Technology, Huazhong Agricultural University, Wuhan, China

**Keywords:** *Picromerus lewisi*, predatory stink bug, neuropeptide, neuropeptide receptor, transcriptome, salivary gland

## Abstract

Predatory stink bugs derive from phytophagous stink bugs and evolved enhanced predation skills. Neuropeptides are a diverse class of ancient signaling molecules that regulate physiological processes and behavior in animals, including stink bugs. Neuropeptide evolution might be important for the development of predation because neuropeptides can be converted to venoms that impact prey. However, information on neuropeptide signaling genes in predatory stink bugs is lacking. In the present study, neuropeptide signaling genes of *Picromerus lewisi*, an important predatory stink bug and an effective biological agent, were comprehensively identified by transcriptome analysis, with a total of 59 neuropeptide precursor genes and 58 potential neuropeptide receptor genes found. In addition, several neuropeptides and their receptors enriched in salivary glands of *P. lewisi* were identified. The present study and subsequent functional research contribute to an in-depth understanding of the biology and behavior of the predatory bugs and can provide basic information for the development of better pest management strategies, possibly including neuropeptide receptors as insecticide targets and salivary gland derived venom toxins as novel killing moleculars.

## 1 Introduction

Neuropeptides are a large diverse class of signaling molecules with key roles in insect physiology and behavior (Schoofs et al., 2017). Neuropeptides mediate their effects mainly through G protein-coupled receptors (GPCRs), receptor guanylyl cyclases (RGCs), and receptor tyrosine kinases (RTKs) (Caers et al., 2012). Given their essential regulatory functions and high specificity, neuropeptide signaling systems have been considered promising targets for “green” pest control (Verlinden et al., 2014; Audsley and Down, 2015). In order to develop a “green” insecticide based off this system, scientists should seek out unique neuropeptide signaling pathways that are absent in beneficial predatory insects. To this end, recent improvements in genome, transcriptome and proteome analysis have led to the discovery of neuropeptides and their receptors in a number of insects, including several beneficial species, which will provide valuable information for the development of novel insecticides with high selectivity.

Heteropteran insects (true bugs) are an ideal group to explore the evolution of trophic strategies (Walker et al., 2016). Heteropterans have diverse feeding strategies. The group includes phytophages (such as Pentatomomorpha and Miridae), entomophages (such as Nepomorpha, Enicocephalomorpha, Leptopodomorpha, Gerromorpha, and Dipsocoromorpha), and hematophages (such as Triatominae and Cimicidae) (Walker et al., 2016; Walker et al., 2018). Entomophagy is also present in some Pentatomomorpha and Miridae groups, with a reversal to predation from phytophagy (Walker et al., 2016). To date, sets of neuropeptides have been analyzed in several heteropterans, including three phytophagous bugs, *Lygus hesperus* (Miridae) (Christie et al., 2016; Hull et al., 2021), *Nezara viridula* (Pentatomidae) and *Halyomorpha halys* (Pentatomidae) (Lavore et al., 2018), and four hematophagous Reduviidae bugs, *Rhodnius prolixus*, *Triatoma dimidiata*, *T. infestans*, and *T. pallidipennis* (Ons et al., 2011; Ons et al., 2016). Comprehensive identification of neuropeptide receptors has only been reported for five hematophagous bugs, *R. prolixus*, *T. dimidiata*, *T. pallidipennis*, *T. infestans* (Ons et al., 2016), and *Cimex lectularius* (Benoit et al., 2016), and two phytophagous bugs, *N. viridula* (Lavore et al., 2018) and *Apolygus lucorum* (Gao et al., 2021). To date, no comprehensive study of neuropeptide signaling genes in predatory heteropterans has been reported. Identifying and comparing neuropeptide sets of diverse bugs is important for finding pest specific targets that do not negatively impact beneficial natural enemies like predatory stink bugs.

The predatory stink bug *Picromerus lewisi* Scott (Hemiptera: Pentatomidae) is widely distributed in China and other Asian regions (Lin et al., 2000). It has been selected as an excellent biological control agent for a wide range of agricultural and forest insect pests, such as Lepidoptera larvae (Mu et al., 2022). Currently, *P. lewisi* has been successfully mass produced by natural enemy factories in China and has been shown to have good control of many important pests, such as *Spodoptera frugiperda* (Wang et al., 2019b; Tang et al., 2019). Predatory stink bugs use their salivary venom to paralyze prey and initiate extra-oral digestion. The salivary glands with venom components (also called venom glands) of predatory stink bugs are likely to be derived from the salivary glands of related non-venomous stink bug species (Cohen, 1990; Cohen, 1995; Walker et al., 2016). Insect salivary systems are influenced by neuropeptides and neurohormones secreted from endocrine organs and various neuronal cells (Spit et al., 2012). For example, in the cockroach, *Periplanea americana*, the SMYamide neuropeptide gene is specifically expressed in the neurons innervating the salivary glands and functions as a hormone to generate action potentials during feeding (Veenstra, 2020). In addition, insect salivary glands also have endocrine functions, producing neuropeptides and neurohormones to regulate other tissues and organs (Li et al., 2022b; Titos et al., 2023). A salivary gland-derived peptide, Sgsf, has been identified in *Drosophila* as an endocrine factor secreted into the hemolymph that systemically regulates larval growth (Li et al., 2022b). What’s more, there are a few interesting cases where invertebrate neuropeptides have been recruited into salivary glands as venom toxins to affect prey, such as TKs from the cephalopods *Eledone moschata* and *Octopus vulgaris* (Champagne and Ribeiro, 1994; Kanda et al., 2003).

Given the state of the field described above, our fundamental hypotheses are that: 1) Insect neuropeptides might be repurposed into novel insecticides; 2) finding a neuropeptide pathway that is different or absent in beneficial predators, could lead to insecticides that do not hurt beneficial predators; 3) natural enemy evolution might repurpose neuropeptides as venom toxins, causing them to be expressed in salivary glands. To begin testing these hypotheses, we identified neuropeptides and their receptors in *P. lewisi* using transcriptome data analysis. In addition, the expression patterns of these genes in heads, guts and salivary glands were analyzed. The fundamental genetic information of the neuropeptide signaling system will be helpful to better understand the biology and ecology of the predatory bugs and to develop better pest management strategies utilizing bothe natural enemies and novel synthetic chemistries.

## 2 Materials and methods

### 2.1 *De novo* assembly of the *P. lewisi* transcriptome

In our previous study, full-length transcriptome and RNA-seq transcriptome analysis of *P. lewisi* were jointly performed (Li et al., 2022a). However, no more than 20 neuropeptide precursor transcripts were found in the full-length transcriptome, which might be because a number of short precursor transcripts were polished during sequence clustering of raw reads obtained from Iso-seq sequencing. Herein, for the identification of neuropeptide signaling genes of *P. lewisi*, we performed *de novo* re-assembly of the RNA-seq transcriptome data that were generated from the previous study (Li et al., 2022a). Briefly, 100 fifth-instar nymphs starved for about 6 h were sampled for total RNA extraction from salivary glands (SG), guts (G), antennae (A), legs (L), and heads without antennae and salivary glands (H), with three independent biological replicates for each tissue/part sample. RNA samples were extracted using TRIzol reagent (Invitrogen, USA) and about 3 μg RNA per sample was used as the template for cDNA synthesis and Illumina sequencing using Ilumina NovaSeq 6000 (Illumina, USA). A total of fifteen libraries with approximately 20 million sequence clean reads each were generated and deposited in the National Center for Biotechnology Information (NCBI) Short Read Archive (SRA) under accession numbers of SRR20681617∼SRR20681631 (Wang et al., 2019a). All libraries were pooled and then assembled into unigenes using the Trinity software (v2.6.6), with min_kmer_cov set to 2 by default and all other parameters set default. A Benchmarking Universal Single-Copy Orthologs (BUSCO) analysis was performed to assess the completeness of the assembly (Simão et al., 2015). All unigenes were *de novo* annotated against Nr/Nt (NCBI non-redundant protein/nucleotide sequences), Pfam (Protein family), KOG/COG (Clusters of Orthologous Groups of proteins), Swiss-Prot (A manually annotated and reviewed protein sequence database), KEGG (Kyoto Encyclopedia of Genes and Genomes), and GO (Gene Ontology) databases.

### 2.2 Differential expression analysis

Differential expression analysis of transcripts from SG, G and H with three biological replicates each was performed using the DESeq2 R package (1.20.0). The Fragments per kilobase of transcript per million fragments mapped (FPKM) value was calculated to estimate the expression level of each transcript from each library. The Benjamini-Hochberg approach was used to adjust *p* values (P_adj) to control the false discovery rate. Genes with P_adj values <0.05 and | log2 (fold change) | > 1 were designated as differentially expressed genes (DEGs). Heat maps showing gene expression profiles were constructed based on Log10 (FPKM +1) values.

To validate the transcriptome data, qRT-PCR analysis of eighteen genes had been performed (Li et al., 2022a). Briefly, eight cytochrome P450 monooxygenase (CYP) genes, six carboxyl/choline esterase (CCE) genes, and four glutathione S-transferase (GST) genes were selected, and the EF1A gene was used as the candidate reference gene. The RNA samples for qRT-PCR were the same as those for RNA-seq sequencing. Significant consistency was found between the expression profiles obtained by qRT-PCR and RNA-Seq (Li et al., 2022a).

### 2.3 Identification of neuropeptides and their receptors

Local tBLASTn searches were performed to predict the genes of neuropeptide precursors and their receptors from the reassembled *P. lewisi* transcriptome. Amino acid sequences of known neuropeptide precursors from *H. halys* (Lavore et al., 2018), *L. hesperus* (Hull et al., 2021), *Nilaparvata lugens* (Tanaka et al., 2014), and *R. prolixus* (Ons et al., 2011; Ons et al., 2016) were used as reference queries. Sequences of neuropeptide GPCR receptors from *N. lugens* (Tanaka et al., 2014), *N. viridula* (Lavore et al., 2018), *A. lucorum* (Gao et al., 2021), and *R. prolixus* (Ons et al., 2016) were collected. Neuropeptide RGC and RTK receptor sequences from *Drosophila melanogaster* and *Aedes aegypti* were obtained from Kong et al. (2021). What’s more, sequences of some potential novel neuropeptide precursors, agatoxin-like peptide (ALP), *Carausius* neuropeptide-like precursor (CNP), parathyroid hormone (PTH), PaOGS36577, and RFLamide (RFLa), were identified based on their homologous genes from *Periplaneta americana* (Zeng et al., 2021) or *Tribolium castaneum* (Xie et al., 2020). In addition, sequences of few novel identified insect neuropeptide receptors, for PTH (Xie et al., 2020), CNMamide (CNMa) (Jung et al., 2014) and elevenin (Ele) (Uchiyama et al., 2017), were also used as reference queries. The BLAST E-value threshold for neuropeptides was 1.0, and the hits were manually checked based on the characteristics of putative mature active peptide sequences. The E-value threshold for receptors was 10^−5^. Besides, the remaining neuropeptide sequences not included in our custom *P. lewisi* transcriptome database, were also searched using a public Sequence Read Archive (SRA) database (SRR10134979) via the NCBI tBLASTn program.

### 2.4 Peptide structural prediction

A well-established workflow was used to predict the potential active peptides of *P. lewisi*. Briefly, the presence of signal peptides was predicted using the online program SignalP 5.0 (https://services.healthtech.dtu.dk/services/SignalP-5.0/). Prohormone cleavage sites and post-translational modifications were identified based on the information presented in Veenstra (Veenstra, 2000) and/or by homology to known arthropod peptides.

### 2.5 Sequence alignment and phylogenetic analysis

Multiple alignments of amino acid sequences were performed using the online program MAFFT version 7 (https://mafft.cbrc.jp/alignment/server/, “G-INS-1” progressive method setting). For the alignments of neuropeptides, the putative active peptides or the amino acid sequences removing the putative signal peptides were adopted. For the alignments of neuropeptide receptors, full-length sequences were used when available, or partial sequences were used. Amino acid identity was subsequently determined and the alignment result was visualized using GeneDoc version 2 (Nicholas et al., 1997). Sequence logos of the aligned peptide sequences were generated using the online program WebLogo (http://weblogo.berkeley.edu/logo.cgi) (Crooks et al., 2004).

Maximum likelihood (ML) phylogenetic analysis was conducted using IQ-TREE (Minh et al., 2020). In a first run, the ModelFinder function was employed to determine the best-fit model using Bayesian Information Criterion (Darriba et al., 2019). In a second run, ML phylogenetic tree was constructed using a ultrafast (UF) bootstrap test with 3000 replicates and default settings to reduce overestimation of bootstrap support (-bnni) (Hoang et al., 2017). Phylogenetic trees were visualized using the Interactive Tree Of Life (iTOL) web server (https://itol.embl.de/) (Letunić and Bork, 2021).

### 2.6 Cloning neuropeptide transcripts

Two species-specific neuropeptide transcripts, Crustacean cardioactive peptide (CCAP) and Orcokinin B (OKB), were amplified by PCR and sequenced to verify their reliability. Primers were designed to amplify the coding sequences (Supplementary Table S1). Five fifth-instar nymphs of *P. lewisi* used for cloning were supplied by the Fenggang County Natural Enemy Breeding Center of the Guizhou Tobacco Company Zunyi Branch, Zunyi, Guizhou Province, China. Total RNA was isolated using TRIzol reagent (Invitrogen, United States). RNA quality and quantity were determined using the RNA Nano 6000 Assay Kit in the Bioanalyzer 2,100 system (Agilent Technologies, United States). Total RNA was treated with DNase I (Invitrogen, United States) to remove any residual genomic DNA. cDNA were synthesized from 1 μg of the total RNA using a SMARTer PCR cDNA Synthesis Kit (Takara Bio United States, Inc, United States). Neuropeptide precursor transcripts were amplified in a 50-uL reaction system using 2 × Phanta Flash Master Mix (Vazyme, China). The PCR procedure was set as follows: pre-denaturation at 98°C for 30 s, 35 cycles of 98°C for 10 s, 55°C for 5 s and 72°C for 5 s, and termination at 72°C for 1 min. The PCR products were cut from the gel, purified and sequenced by Beijing Tsingke Biotech Co., Ltd.

## 3 Results

### 3.1 Transcript assembly

The raw sequences of 15 biosamples from five tissues/parts with three biological repeats each were re-assembled using the *de novo* assembly procedure, resulting in a total of 62,183 unigenes with a mean length of 1,203 bp, and an N50 length of 2,040 bp (Supplementary Table S2). BUSCO analysis showed a high degree of completeness (87.0%) in the assembly (Supplementary Figure S1). 41.9% of the assembled unigenes were annotated in at least one database and 64.1% of the annotated unigenes were best matched to their *H. halys* homologs (Supplementary Table S2).

### 3.2 Identification of neuropeptide precursors in *P. lewisi*

**FIGURE 1:**
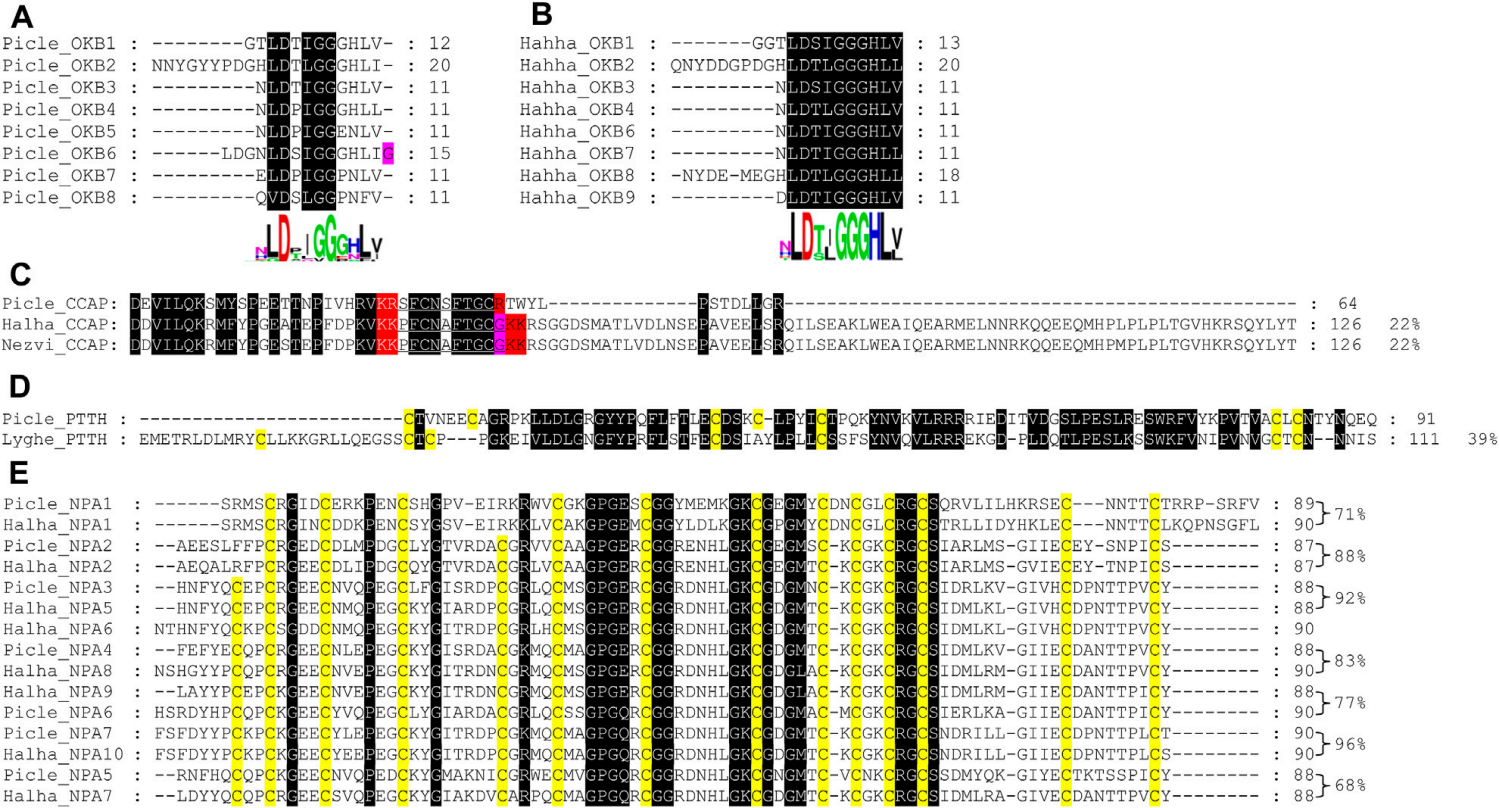
Multiple sequence alignment of four neuropeptides from *P. lewisi* and other bugs. **(A,B)** OKB active peptides from *P. lewisi* and *H. halys*. **(C)** CCAP precursors cutting signal peptides from *P. lewisi*, *H. halys* and *N. viridula*. **(D)** PTTH precursors cutting signal peptides from *P. lewisi* and *L. hesperus*. **(E)** NPA precursors cutting signal peptides from *P. lewisi* and *H. halys*. Full conservative residues are shaded in black background. Predicted convertase cleavage sites are shaded in red. Glycine residues are shaded in pink. Conserved cysteine residues are shaded in yellow. Identity values between the *P. lewisi* precursor and its ortholog are shown at the end.

A total of 59 neuropeptide precursors were identified in the *P. lewisi* transcriptome, including several novel neuropeptides, CNP, PTH, PaOGS36577, and RFLa (Table 1; Supplementary Data S1). Glycoprotein hormone beta 5 (GPB5), allatostatin C (AST-C), and trissin were not found in the *P. lewisi* transcriptome. Although AST-C was not identified, potential homologs of AST-CC and AST-CCC were found in *P. lewisi* and other hemipterans (Table 2). GPB5 was identified from *H. halys* and *R. prolixus* but still not found in *N. viridula* and *L. hesperus* (Table 2). Adipokinetic hormone (AKH) and sulfakinin (SK) were not found in our custom transcriptome but were identified in NCBI SRA data (SRR10134979). 50 neuropeptide transcripts have full-length sequences and the remaining nine non-full-length sequences include AKH, ALP1, ALP2, arginine-vasopressin-like peptide (AVLP), natalisin (NTL), OKA, OKB, PTH and SK (Supplementary Data S1). Most of the predicted *P. lewisi* active neuropeptides are identical or highly similar to their homologous peptides from *H. halys* or other bugs (Table 1), whereas a few peptides have low similarity to their homologs, such as OKB, CCAP, prothoracicotropic hormone (PTTH) and neuroparsin A5 (NPA5) (with no more than 70% identity) (Figure 1). To increase the confidence in the putative *P. lewisi*-specific neuropeptide precursors, the coding sequences of the OKB and CCAP transcripts were targeted for RT-PCR amplification. Amplimers of the expected sizes were obtained for these two transcripts (730 bp and 195 bp for OKB and CCAP, respectively; Supplementary Figure S1). All of the cloned products were found to have 100% nucleotide identity with the *in silico* sequences from the transcriptomic data.

**TABLE 1:** Putative neuropeptides precursor genes identified from *Picromerus lewisi*.

Neuropeptide	Acronym	Types^a^	Homologous gene	Identity^b^	H	G	SG
Adipokinetic hormone	AKH^c^	S1	NW_020110344.1 [*H. halys*]	100%			
Agatoxin-like 1	ALP1	L1	XP_014292753.1 [*H. halys*]	100%			
Agatoxin-like 2	ALP2	L1	XP_014292752.1 [*H. halys*]	100%			
AKH/Corazonin-relate peptide	ACP	S1	XP_014285630.1 [*H. halys*]	100%			
Allatostatin A/FGLamide Allatostatin	AST-A	S6	XP_014282383.1 [*H. halys*]	75%∼100%			
Allatostatin B/Myoinhibitory peptide	AST-B	S13(S10)	XP_024219541.1 [*H. halys*]	73%∼100%			
Allatostatin CC	AST-CC	S1	XP_014284063.1 [*H. halys*]	100%		*	
Allatostatin CCC	AST-CCC	S1	NW_020113761 [*H. halys*]	100%			
Allatotropin	AT	S1	XP_014274846.1 [*H. halys*]	100%			
Arginine-vasopressin-like peptide	AVLP	S1	XP_014287933.1 [*H. halys*]	100%			
Bursicon alpha	Burα	L1	XP_014275825.1 [*H. halys*]	98%			
Bursicon beta	Burβ	L1	XP_024214523.1 [*H. halys*]	98%			
Capability/Cardio acceleratory peptide 2b	CAPA	S3	AYP97817.1 [*H. halys*]	90%∼100%			
*Carausius* neuropeptide-like precursor	CNP	S?^d^	XP_024214992.1 [*H. halys*]	100%			
CCHamide 1	CCHa1	S1	XP_014293977.1 [*H. halys*]	100%			
CCHamide 2	CCHa2	S1	AZK31334.1 [*N.viridula*]	100%		*	
CNMamide	CNMa	S1	XP_024219068.1 [*H. halys*]	93%		*	
Corazonin	Crz	S1	XP_014274138.1 [*H. halys*]	100%			
Crustacean Cardioactive peptide	CCAP	S1	XP_014284775.1 [*H. halys*]	70%			*
Diuretic hormone 31	DH31	L1	XP_024214033.1 [*H. halys*]	100%			
Diuretic Hormone 44	DH44	L1	XP_014283173.1 [*H. halys*]	93%			
Ecdysis triggering hormone	ETH	S3	XP_014275716.1 [*H. halys*]	100%			
Eclosion hormone 1	EH1	L1	XP_024214295.1 [*H. halys*]	100%			
Eclosion hormone 2	EH2	L1	BAV78806.1 [*P. stali*]	98%			
Elevenin	Ele	S1	XP_024216787.1 [*H. halys*]	100%			
FMRFamide	FMRFa	S7	XP_024219218.1 [*H. halys*]	92%∼100%			
Glycoprotein hormone alpha 2	GPA2	L1	XP_014286664.1 [*H. halys*]	92%			
IDLSRF-like	IDLSRF	S1	QQN72879.1 [*L. hesperus*]	100%			
Insulin-like peptide 1	ILP1	L2	XP_014280184.1 [*H. halys*]	92%∼96%			
Insulin-like peptide 2	ILP2	L2	XP_024216518.1 [*H. halys*]	94%∼100%			
Ion transport peptide	ITP	L1	XP_014274475.1 [*H. halys*]	100%			
ITG-like	ITG	S1	XP_014275756.1 [*H. halys*]	89%			
Leucokinin	LK	S15	XP_014275383.1 [*H. halys*]	87%∼100%			
Myosuppressin	MS	S1	XP_024214034.1 [*H. halys*]	100%			
Natalisin	NTL	S1	NW_020111212.1 [*H. halys*]	94%			
Neuroparsin A1	NPA1	L1	XP_014291036.1 [*H. halys*]	71%			
Neuroparsin A2	NPA2	L1	XP_014291032.1 [*H. halys*]	88%			
Neuroparsin A3	NPA3	L1	XP_014279505.1 [*H. halys*]	92%			
Neuroparsin A4	NPA4	L1	XP_014279508.1 [*H. halys*]	83%			
Neuroparsin A5	NPA5	L1	XP_014279502.1 [*H. halys*]	68%			
Neuroparsin A6	NPA6	L1	XP_014279507.1 [*H. halys*]	77%			
Neuroparsin A7	NPA7	L1	XP_014279506.1 [*H. halys*]	96%			
Neuropeptide F	NPF	L1	XP_014289207.2 [*H. halys*]	100%			
Neuropeptide-like precursor 1	NPLP1	S?^d^	XP_014276590.1 [*H. halys*]	84%			
NVP-like	NVP	S4	XP_014289776.1 [*H. halys*]	93%∼100%			
Orcokinin A	OKA	S3	XP_014280358.1 [*H. halys*]	100%			
Orcokinin B	OKB	S8	XP_014280359.2 [*H. halys*]	45%∼100%		*	*
PaOGS36577	PaOGS36577	S1	XP_014283619.2 [*H. halys*]	100%			
Parathyroid hormone	PTH	L1	XP_014293861.1 [*H. halys*]	97%			
Pigment dispersing factor	PDF	S1	XP_024215414.1 [*H. halys*]	100%			
Proctolin	Pro	S1	XP_014283232.1 [*H. halys*]	100%			
Prothoracicotropic hormone	PTTH	L1	QQW38907.1 [*L. hesperus*]	39%			
Pyrokinin	PK	S3	AYP97818.1 [*H. halys*]	90%∼100%			
RFLamide	RFLa	S1	XP_014286502.1 [*H. halys*]	100%			
RYamide	RYa	S3	XP_014276031.1 [*H. halys*]	100%			
Short Neuropeptide F	sNPF	S1	XP_014284284.1 [*H. halys*]	100%			
SIFamide	SIFa	S1	XP_024214831.1 [*H. halys*]	100%			
Sulfakinins	SK^c^	S2	XP_014274494.1 [*H. halys*]	91%∼100%			
Tachykinins	TK	S7	XP_024216981.1 [*H. halys*]	100%			

^a^ Neuropeptides from *P. lewisi* are classified into those encoding a single and short active peptide (S1, the length of an encoded peptide is less than 25 amino acid residues), multiple and short active peptides (Sx, x as the number of encoded peptides, the number in parentheses indicates the distinct peptide numbers), and long active peptides (L1∼L2, the length of an encoded peptide is more than 25 amino acid residues).

^b^ Identity values of predicted active peptides of *P. lewisi* and their homologous peptides were calculated based on pairwise alignemnts or multiple alignments.

^c^ Expression levels of two neuropeptide genes (AKH, and SK) are missing because they were identified from the public SRA, database.

^d^ The potential short active peptides of CNP, and NPLP1 of *P. lewisi* are not clear.

*indicates that the expression level of a gene was significantly higher in G or SG than that in H, with log2 (fold) >1 and P_adj<0.05.

The heatmap scale among heads (H), guts (G) and salivary glands (SG) was based on Log10 (FPKM+1) values:
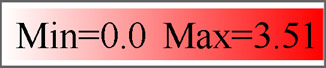

**TABLE 2:** Neuropeptide precursors identified in *P. lewisi* and four other heteropteran species.

Species	*P. lewisi*	*H. halys*	*N. viridula*	*L. hesperus*	*R. prolixus*
Family	Pentatomidae	Pentatomidae	Pentatomidae	Miridae	Reduviidae
Feeding habit	Predaceous	Phytophagous	Phytophagous	Phytophagous	Hematophagous
AKH	+	+	+	+	+
Crz	+	+	+	+	+
ACP	+	+	+	+	+
ALP	2	2	Not Found	+	Not Found
AVLP	+	+	+	Not Found	Not Found
ASTA	+	+	+	+	+
ASTB	+	+	+	+	+
ASTC	Not Found	Not Found	Not Found	Not Found	Not Found
ASTCC	+	+	+	+	+
ASTCCC	+	+	+	+	+
AT	+	+	+	+	+
Burα	+	+	+	+	+
Burβ	+	+	+	+	+
CAPA	+	+	+	+	+
CCAP	+	+	+	+	+
CCHa1	+	+	+	+	+
CCHa2	+	+	+	+	+
CNMa	+	+	+	+	+
CNP	+	+	+	Not Found	Not Found
DH31	+	+	+	+	+
DH44	+	+	+	+	Not Found
EH1	+	+	Not Found	+	+
EH2	+	+	Not Found	+	Not Found
Ele	+	+	+	+	+
ETH	+	+	+	+	+
FMRFa	+	+	+	+	+
GPA2	+	+	+	Not Found	+
GPB5	Not Found	+	Not Found	Not Found	+
IDLSRF	+	Not Found	+	+	Not Found
ILP	2	2	+	3	4
ITG	+	+	+	Not Found	+
ITP	+	+	+	2	+
LK	+	+	+	+	+
MS	+	+	+	+	+
NPA	7	12	13	4	+
NPF	+	+	+	+	+
NPLP1	+	+	+	+	+
NTL	+	+	Not Found	+	+
NVP	+	+	+	+	+
OKA	+	+	+	+	3
OKB	+	+	2	+	+
PaOGS36577	+	+	Not Found	Not Found	Not Found
PDF	+	+	+	+	+
Pro	+	+	+	+	+
PTH	+	+	Not Found	Not Found	Not Found
PTTH	+	Not Found	Not Found	+	Not Found
PK	+	+	Not Found	+	+
RFLa	+	+	Not Found	Not Found	Not Found
RYa	+	+	+	+	+
SIFa	+	+	+	+	+
sNPF	+	+	+	+	+
SK	+	+	+	+	+
TK	+	+	+	+	+
Trissin	Not Found	Not Found	Not Found	Not Found	Not Found

“+”: identified with the single precursor gene. Numbers in grids indicate the number of multiple precursor genes identified. Data for other species were mainly taken from *H. halys* (Lavore et al., 2018), *N. viridula* (Lavore et al., 2018), *L. hesperus* (Hull et al., 2021) and *R. prolixus* (Ons et al., 2011; Ons et al., 2016).

The *P. lewisi* OKB precursor can encode eight potential active peptides that share the DXI/LGGG consensus sequence (Figure 1A). The *H. halys* OKB precursor can also encode eight distinct active peptides. Only one of the *P. lewisi* OKB active peptides shares the identical sequence (NLDTIGGGHLV) with an *H. halys* OKB peptide, whereas other *P. lewisi* OKB peptides had 45%∼90% identity with their *H. halys* orthologues (Figures 1A∼1B).

CCAP is known as the most conserved arthropod neuropeptide, with an identical amino acid sequence, PFCNAFTGCamide, found in all insects examined. Unexpectedly, the mature CCAP peptide predicted from *P. lewisi* (SFCNSFTGC) has two variant residues and lacks a C-terminal glycine residue (Figure 1C). The same CCAP peptide sequence was also found in *P. lewisi* RNA-seq SRA reads released in NCBI (e.g., SRR10134979.5654305.2).

The *P. lewisi* PTTH transcript has features typical of other insect PTTHs, encoding a putative active peptide containing seven cystine residues that form inter- and intra-chain disulfide bonds. PTTH has also been found in another heteropteran species, *L. hesperus*, but it is absent in a number of heteropteran species such as *H. halys*, *N. viridula*, and *R. prolixus* (Table 2). The *P. lewisi* PTTH peptide shares 39% amino acid identity with that of *L. hesperus* (Figure 1D).

A total of seven *P. lewisi* NPA transcripts were found (Supplementary Data S1). The *P. lewisi* NPA precursors can encode potential active peptides with a length of approximately 80 amino acid residues and 12∼14 cystine residues forming 6∼7 intrachain disulfide bridges (Figure 1E). Sequence alignment of the NPA peptides showed that Picle_NPA5 shares a relatively low identity (68%) with its *H. halys* orthologue, whereas other *P. lewisi* NPA peptides had 71%∼96% identity with their *H. halys* orthologs (Figure 1E).

### 3.3 Identification of salivary gland-specific neuropeptides in *P. lewisi*

A heatmap based on FPKM values of 57 neuropeptide genes (excluding AKH and SK, which were identified from the NCBI SRA database) in heads, salivary glands, and guts of *P. lewisi*, is shown in Table 1. A total of 22 neuropeptide genes were found to be expressed in salivary glands using a criterion of more than 1 FPKM in at least one repeat (Supplementary Table S3). Among them, six neuropeptides, OKB, CCAP, OKA, diuretic hormone 31 (DH31), ALP2, and ecdysis triggering hormone (ETH), showed FPKM values higher than ten in at least one repeat (Supplementary Table S3).

Compared to heads, only two neuropeptide genes (OKB and CCAP) were significantly more highly expressed in salivary glands, with log2 (fold) >1 and P_adj <0.05 (Table 1). OKB had the highest FPKM value (3228.1) in the salivary glands among all the identified neuropeptide genes and its relative expression level in the salivary glands compared to heads was 190.6-fold. CCAP was the second most highly expressed neuropeptide gene in the salivary glands (FPKM value = 109.3) and it was also more highly expressed in the salivary glands than heads, with an expression fold of 134.9. OKB, CNMa, AST-CC and CCHamide 2 (CCHa2) were significantly upregulated in guts than heads of *P. lewisi* (Table 1).

### 3.4 Identification of neuropeptide receptors in *P. lewisi*

**FIGURE 2:**
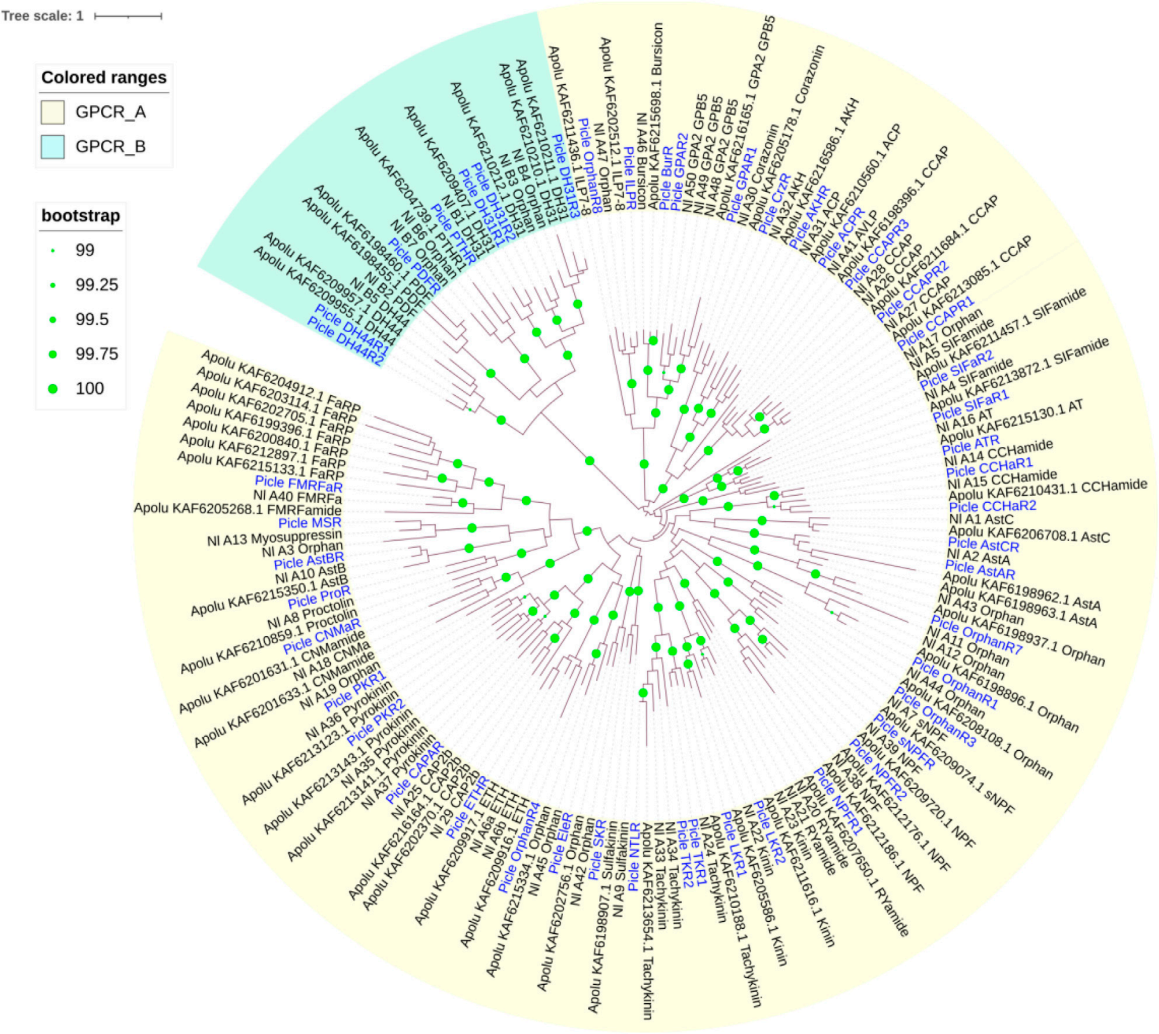
Phylogenetic tree of neuropeptide GPCR receptors from *P. lewisi* (gene IDs beginning “Picle”), *A*. *lucorum* (Apolu) and *N. lugens* (Nl). The ultrafast (UF) bootstrap value with more than 99% was marked in the tree. The *P. lewisi* gene names are marked with a blur color.

**FIGURE 3:**
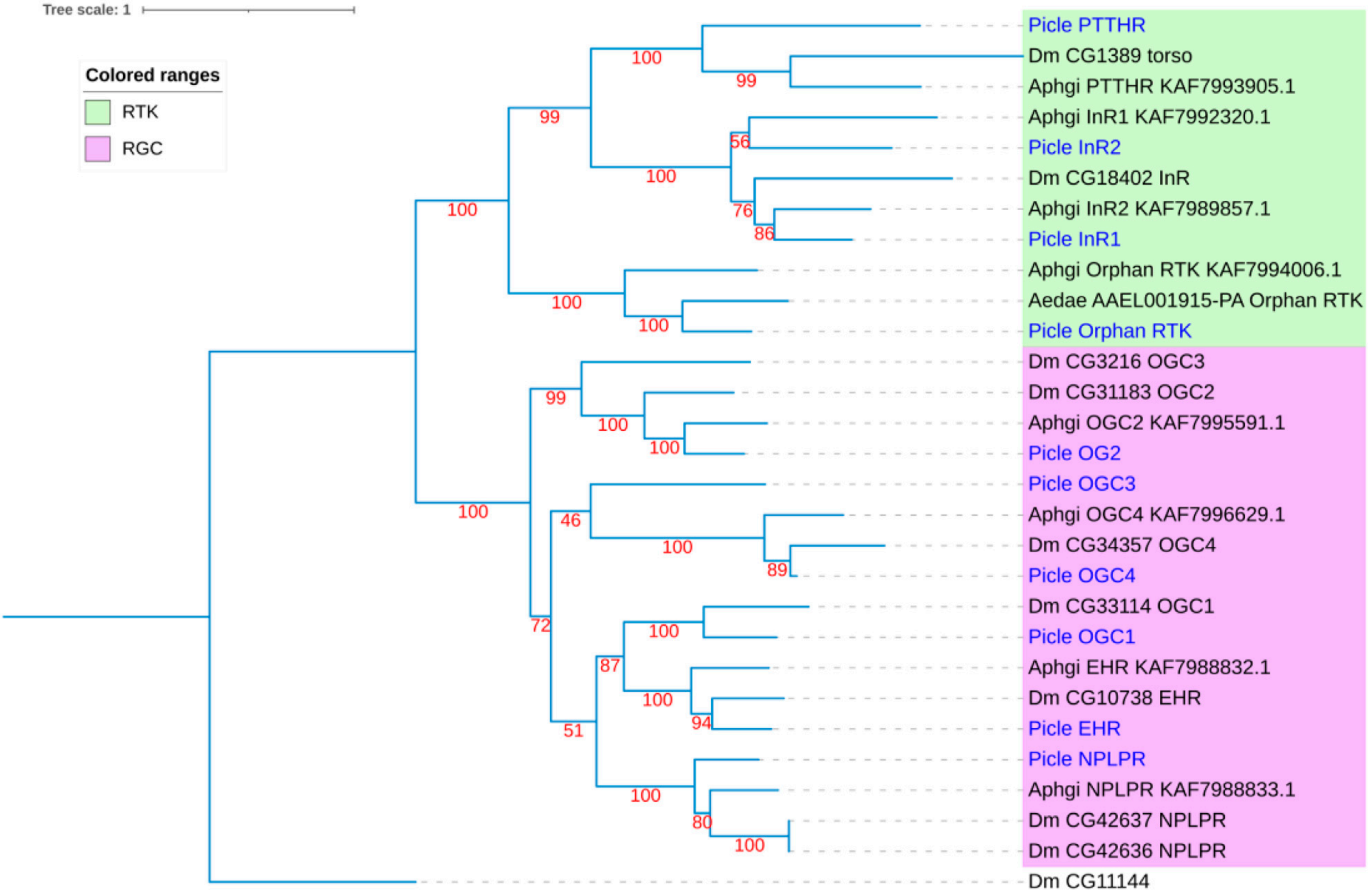
Phylogenetic tree of neuropeptide receptor guanylyl cyclases (RGCs) and receptor tyrosine kinases (RTKs) from *P. lewisi* (gene IDs beginning with “Picle”), *D*. *melanogaster* (Dm), *Aphidius gifuensi* (Aphgi), and *Aedes aegypti* (Aedae). The UF bootstrap value is marked in the tree. The *P. lewisi* gene names are marked with a blue color.

Using homology to research against our transcriptome data producted a total of 58 potential neuropeptide receptor genes (Table 3; Supplementary Data S2), including 41 family A GPCRs (rhodopsin-like receptors), seven family B GPCRs (secretin-like receptors), six RGCs and four RTKs. Eighteen neuropeptide receptor transcripts were partial sequences and the remaining 40 were full-length (Table 3; Supplementary Data S2). The family A neuropeptide GPCRs of *P. lewisi* can be classified into 27 groups based on their putative ligands: Receptors for AKH, AKH/corazonin-related peptide (ACP), AST-C, allatotropin (AT), CCAP, CNMa, corazonin (Crz), Ele, myosuppressin (MS), NTL, proctolin (Pro), SIFamide (SIFa), Short neuropeptide F (sNPF), SK, capability/cardioacceleratory peptide 2b (CAPA), ETH, pyrokinin (PK), AST-A, FMRFamide (FMRFa), tachykinins (TKs), AST-B, leucokinin (LK), bursicon (Bur), GPA2/GPB5, neuropeptide F (NPF), insulin-like peptide (ILP), and orphan (Table 3; Figure 2). No orthologous gene encoding the receptors for AVLP, RYamide (RYa) or trissin was identified in the *P. lewisi* transcriptome. The family B neuropeptide GPCRs of *P. lewisi* can be subdivided into four groups: receptors for DH31, DH44, pigment dispersing factor (PDF) and PTH (Table 3; Figure 2). Among RGCs, eclosion hormone receptor (EHR), Neuropeptide-like precursor receptor (NPLPR), and four orphan RGCs were found in *P. lewisi* (Table 3; Figure 3). In addition to RTKs, one PTTH receptor (PTTHR), two insulin-like peptide receptors (InR1 and InR2) and one orphan RTK receptor were identified (Table 3; Figure 3).

**TABLE 3:** Neuropeptides receptor genes identified from *Picromerus lewisi*.

Gene name	Class	Predicted TMHs	Homologous gene	E value	Identity (%)^a^	H	G	SG
Picle_AKHR^b^	GPCR_A	7	Nl_A32_AKH [*N. lugens*]	-	-			
Picle_ACPR	GPCR_A	4^c^	Nl_A31_ACP [*N. lugens*]	1E-68	53			
Picle_AstAR	GPCR_A	7^c^	Nl_A2_AST-A [*N. lugens*]	1E-157	74			
Picle_AstBR	GPCR_A	7	Nl_A10_AST-B [*N. lugens*]	0	82			
Picle_AstCR	GPCR_A	7	Nl_A1_AST-C [*N. lugens*]	1E-151	84			
Picle_ATR	GPCR_A	5^c^	Nl_A16_AT [*N. lugens*]	1E-86	64			
Picle_BurR	GPCR_A	5^c^	Nl_A46_Bur [*N. lugens*]	0	67			
Picle_CAPAR	GPCR_A	7	Nl_A25_CAP2b [*N. lugens*]	1E-125	59		*	*
Picle_CCAPR1	GPCR_A	7	Nl_A27_CCAP [*N. lugens*]	1E-146	81			
Picle_CCAPR2	GPCR_A	7	Nl_A26_CCAP [*N. lugens*]	1E-126	60			
Picle_CCAPR3	GPCR_A	7	Nl_A28_CCAP [*N. lugens*]	1E-143	66			
Picle_CCHaR1	GPCR_A	5^c^	Nl_A14_CCHa [*N. lugens*]	1E-119	67			
Picle_CCHaR2	GPCR_A	7	Nl_A15_CCHa [*N. lugens*]	1E-134	64			
Picle_CNMaR	GPCR_A	7	Nl_A18_CNMa [*N. lugens*]	1E-91	44			
Picle_CrzR	GPCR_A	7	Nl_A30_Crz [*N. lugens*]	1E-114	70			
Picle_ETHR	GPCR_A	6^c^	Nl_A6_ETH [*N. lugens*]	1E-132	63			
Picle_FMRFaR	GPCR_A	7	Nl_A40_FMRFa [*N. lugens*]	1E-140	62			
Picle_GPAR1	GPCR_A	7^c^	Nl_A48_GPA2/GPB5 [*N. lugens*]	1E-119	62		*	
Picle_GPAR2	GPCR_A	7	Nl_A49_GPA2/GPB5 [*N. lugens*]	0	56		*	*
Picle_ILPR	GPCR_A	7^c^	Nl_A47_Orphan [*N. lugens*]	1E-168	72			
Picle_LKR1	GPCR_A	7	Nl_A22_Kinin [*N. lugens*]	1E-131	64			
Picle_LKR2	GPCR_A	7^c^	Nl_A23_Kinin [*N. lugens*]	1E-148	67			
Picle_MSR	GPCR_A	7	Nl_A13_MS [*N. lugens*]	1E-140	66			
Picle_NPFR1	GPCR_A	7	Nl_A39_NPF [*N. lugens*]	1E-116	63			
Picle_NPFR2	GPCR_A	7	Nl_A38_NPF [*N. lugens*]	1E-121	66			
Picle_OrphanR1	GPCR_A	7	Nl_A12_Orphan [*N. lugens*]	1E-138	66			
Picle_OrphanR2	GPCR_A	4^c^	Nl_A44_Orphan [*N. lugens*]	1E-56	67			
Picle_OrphanR3	GPCR_A	3^c^	Nl_A45_Orphan [*N. lugens*]	1E-46	60			
Picle_EleR	GPCR_A	2^c^	Nl_A42_Ele [*N. lugens*]	1E-36	63			*
Picle_OrphanR4	GPCR_A	7^c^	Nl_A43_Orphan [*N. lugens*]	1E-82	47			
Picle_OrphanR5	GPCR_A	0^c^	Nl_A47_Orphan [*N. lugens*]	1E-153	56			
Picle_PKR1	GPCR_A	7	Nl_A36_PK [*N. lugens*]	1E-135	57			
Picle_PKR2	GPCR_A	7	Nl_A37_PK [*N. lugens*]	1E-109	63			
Picle_ProR	GPCR_A	7	Nl_A8_Pro [*N. lugens*]	1E-95	51			
Picle_SIFaR1	GPCR_A	7	Nl_A4_SIFa [*N. lugens*]	1E-153	69			
Picle_SIFaR2	GPCR_A	7	Nl_A5_SIFa [*N. lugens*]	1E-127	56			
Picle_SKR	GPCR_A	7	Nl_A9_SK [*N. lugens*]	1E-121	65			
Picle_sNPFR	GPCR_A	7	Nl_A7_sNPF [*N. lugens*]	1E-164	70			
Picle_NTLR	GPCR_A	7^c^	Nl_A33_TK [*N. lugens*]	1E-123	75			
Picle_TKR1	GPCR_A	6^c^	Nl_A24_TK [*N. lugens*]	1E-168	81			
Picle_TKR2	GPCR_A	7	Nl_A34_TK [*N. lugens*]	1E-108	51			
Picle_DH31R1	GPCR_B	7	Nl_B1_DH31 [*N. lugens*]	1E-160	68			
Picle_DH31R2	GPCR_B	7	Nl_B3_Orphan [*N. lugens*]	1E-144	66			
Picle_DH31R3	GPCR_B	7	Nl_B4_Orphan [*N. lugens*]	1E-168	70			
Picle_DH44R1	GPCR_B	7	Nl_B5_DH44 [*N. lugens*]	1E-112	57			
Picle_DH44R2	GPCR_B	7	Nl_B5_DH44 [*N. lugens*]	1E-107	56			*
Picle_PDFR	GPCR_B	7	Nl_B2_PDF [*N. lugens*]	1E-162	65			
Picle_PTHR	GPCR_B	7	Nl_B6_Orphan [*N. lugens*]	1E-110	56			
Picle_EHR	RGC	0	CG10738 [*D. melanogaster*]	0	61			*
Picle_NPLPR	RGC	0	CG42636 [*D. melanogaster*]	0	62		*	*
Picle_OGC1	RGC	1	CG33114 [*D. melanogaster*]	0	56			
Picle_OGC2	RGC	0	CG31183 [*D. melanogaster*]	0	56			
Picle_OGC3	RGC	0^c^	CG3216 [*D. melanogaster*]	1E-48	56			
Picle_OGC4	RGC	0^c^	CG34357 [*D. melanogaster*]	1E-78	84			
Picle_InR1	RTK	1	CG18402 [*D. melanogaster*]	0	37			
Picle_InR2	RTK	1	CG18402 [*D. melanogaster*]	1E-176	32			*
Picle_Orphan_RTK	RTK	2	AAEL001915 [*A*. *aegypti*]	0	58		*	*
Picle_PTTHR	RTK	2	CG1389 [*D. melanogaster*]	1E-44	46			

^a^ Identity values of neuropeptide receptor genes of *P. lewisi* and their homologous genes were calculated based on pairwise alignments or multiple alignments.

^b^ Expression level of AKHR, is missing because it was identified from the public SRA, database.

^c^ Not full length.

*indicates that the expression level of one gene in G or VG was significantly higher than that in H, with log2 (fold) >1 and P_adj<0.05.

The heatmap scale among heads (H), guts (G) and salivary glands (SG) was based on Log10 (FPKM+1) values:
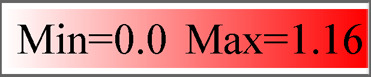

### 3.5 Identification of salivary gland-specific neuropeptide receptor genes in *P. lewisi*

A heatmap based on FPKM values of 57 neuropeptide receptor genes (excluding AKHR, which was identified from the trinity sequences rather than the unigene sequences of our custom transcriptome) in heads, guts and salivary glands is shown in Table 3. A total of 15 out of 57 neuropeptide receptors were expressed in *P. lewisi* salivary glands with the FPKM values higher than one in at least one repeat, among which two receptor genes (EHR and NPLPR) presented the FPKM value higher than 10 in one repeat (Supplementary Table S4). Compared to heads, receptors for EH, NPLP, CAPA, GPA2/GPB5, Ele, DH44, and ILP, and one orphan RTK receptor were more highly expressed in the salivary glands than heads, with log 2 (fold) >1 and P_adj <0.05 (Table 3). Receptors for CAPA, NPLP, and GPA2/GPB5, and one orphan RTK receptor were significantly more highly expressed in guts than heads (Table 3).

## 4 Discussion

In the present study, neuropeptide signaling genes were systematically identified in the predatory stink bug *P. lewisi*, with a total of 59 neuropeptide precursor genes and 58 potential neuropeptide receptor genes found. The number of neuropeptide precursors and their receptors identified in *P. lewisi* is similar to that found in *H. halys* and higher than those in other heteropterans such as *N. viridula*, *L. hesperus*, and *R. prolixus* (Christie et al., 2016; Lavore et al., 2018; Hull et al., 2021). In *P. lewisi*, nearly all of the neuropeptide signaling genes identified in other heteropterans were found, with the exception of GPB5 and RYaR. GPB5 and RYaR were not detected in the *P. lewisi* transcriptome, likely due to sample bias inherent to transcriptomes as opposed to genomes.

Tissue-specific expression profiles of neuropeptides and their receptors in *P. lewisi* provide basic information for in-depth studies of the biology and ecology of this important biological control species. The majority of neuropeptide precursor genes and their receptor genes in *P. lewisi* were found to be predominantly expressed in heads, indicating that most of neuropeptide signaling systems may act mainly as neuroregulators in the predatory bugs and/or derive from tissues in the head. A few neuropeptide signaling genes were also expressed in salivary glands and guts. These may play critical roles in the regulation of feeding and digestion. Neuropeptides in insect guts are involved in the regulation of feeding processes, including food choice, appetite, digestion, metabolism and excretion (Abou El Asrar et al., 2020). DH31, CCHa-1, CCHa-2, NPF, and CNMa are well-known insect neuropeptides associated with the gut-brain axis, regulating appetite, sleep and even courtship (Sano et al., 2015; Kim et al., 2021; Lin et al., 2022; Malita et al., 2022; Titos et al., 2023). In *P. lewisi*, three neuropeptides (CNMa, ASTCC and CCHa2) and five receptor genes were significantly upregulated in the guts when compared to heads, suggesting that they may have functions in feeding regulation.

Multiple neuropeptides and their receptors were identified as genes expressed in insect salivary glands. In the flyAtlas database (https://motif.mvls.gla.ac.uk/FlyAtlas2/), ion transport peptide (ITP), ILP6, NPLP2, NPLP4, and receptors for Crz, GPA2/GPB5, ILPs and NPLP1 were expressed in *Drosophila* salivary glands (Nässel and Zandawala, 2019). DH31, CCAP, CCAPR, AST-B, Pro and NPLP1 were expressed in *R*. *prolixus* salivary glands (Ons, 2017). FMRFa, AstA, SIFa and TK have also been detected in the salivary glands of other insects (Audsley and Weaver, 2009; Veenstra, 2020; Yu et al., 2020). Receptors for PDF, AST-A, DH44, TK, sNPF and DH31 were expressed at higher levels in the salivary glands of the ectoparasitoid, *Habrobracon hebetor* (Yu et al., 2020). In *P. lewisi*, three neuropeptide genes (CCAP and OKB) and eight receptors were enriched in salivary glands. In all these cases, the physiological functions of only a few neuropeptides and their receptors in insect salivary glands have been determined. More research is needed on this topic to describe specific functions.

Several insect neuropeptides have been implicated in the neural control of salivary production or secretion, such as FMRFa from the blow fly *Calliphora vomitoria* (Duve et al., 1992) and the kissing bug *R. prolixus* (Ons, 2017). In *R. prolixus*, AST-B, Pro, CCAP, and CCAPR have also been detected in processes innervating salivary glands, indicating their involvement in the hormonal control of salivary production or secretion (Ons, 2017). In ticks, several neuropeptides have been identified to be expressed in innervations of salivary glands, such as AST-B, DH31, Ele, ELeR, NPLP1, OKA, PDF, SIFa, and SIFaR (Sterkel et al., 2011; Simo et al., 2012; Ladislav et al., 2015; Kim et al., 2018; Vancová et al., 2019; Guerrib et al., 2023). CCAP, OKA, DH31, and NPLPR, which were highly expressed in *P. lewisi*, indicated their possible functions in neural control of the salivary system, as consistent with previous studies, however, expression of OKB and EHR in venom gland or salivary glands has not been reported in other insects so far.

Limited studies provide evidence for the functions of neuropeptides derived from invertebrate salivary glands as endocrine factors that regulate other tissues and organs. In addition, some of these salivary peptides might be venom toxins which alter the physiology of another species. A attractive study demonstrated that a peptide Sgsf expressed in *Drosophila* salivary glands can be secreted into the hemolymph and regulate Dilp2 secretion in the brain (Li et al., 2022b). Nässel et al. (2019) reviewed that TKs produced by salivary glands of mosquitos and cephalopods have been identified as exogenous vasodilators or paralyzing agents that can be delivered to prey. Determining whether neuropeptides expressed in the salivary glands of *P. lewisi* could be endogenous endocrine factors or exogenous venom peptides is important because venom peptides might be utilized as novel insecticidal peptides and/or signaling neuropeptides that their receptors might become useful targets for insecticides.

Our study presented species- and tissue-specific expression patterns of the neuropeptide signaling system in the predatory bugs, which will be used to generate testable functional genetic hypotheses in future studies. The most noteworthy neuropeptide is *P. lewisi* OKB, which was the most highly expressed neuropeptide in *P. lewisi* salivary glands. The expression pattern of OKB in the nervous system and intestine has also been reported in *D. melanogaster*, *Bombyx mori*, *T. castaneum*, *R. prolixus*, and *Blattella germanica* (Chen et al., 2015; Jiang et al., 2015; Ons et al., 2015; Wulff et al., 2017; Wang et al., 2019a). However, expression of OKB in salivary glands has not been reported in other insect species to date. OKs were first discovered with the myotropic activity in the crayfish *Orconectes limosus* (Stangier et al., 1992). One study suggested a role of OKB awakening behavior in *T*. *castaneum* (Ladislav et al., 2015). There is plenty of evidence for the recruitment of neuropeptides into animal venoms (Nässel et al., 2019; Sachkova et al., 2020; Goudarzi et al., 2023). Therefore, we would like to determine whether OKBs could be recruited as venom peptides in the salivary glands of *P. lewisi* and injected into prey to regulate myotropic activity or behavior. From an evolutionary perspective, the high genetic diversity of OKB peptide sequences possibly supports the repurposing of neuropeptides into venom peptides. A well-known example is TKs, another highly diverse and pleiotropic neuropeptide class, which have been convergently recruited into the venom or salivary glands of venomous invertebrates to affect prey (Nässel et al., 2019). This interesting evolutionary path of neuropeptide recruitment for novel toxins has also been recently revealed in the sea anemone *Nematostella vectensis* (ShK-like peptides) and the caterpillar *Acharia stimulea* (RF-amide peptides) (Sachkova et al., 2020; Goudarzi et al., 2023). Nevertheless, we cannot exclude the possibility that OKB peptides are endogenous endocrine factors. The OK receptor has not yet been identified in any species, therefore efforts to identify the OK receptor in insects will be crucial to understanding the role of OKBs in the salivary glands of *P. lewisi*.

The most unexpected result in the present study was finding a very atypical CCAP in *P. lewisi*. To our knowledge, CCAP is the identical neuropeptide (PFCNAFTGC-NH_2_) in all examined insect species. CCAP from *P. lewisi* (SFCNSFTGC) identified in the present study has two variant amino acid residues and no amidation at the C-terminus. Thus our findings indicate a highly novel primary structure for this peptide which we will seek to confirm through further studies utilizing liquid chromatography tandem mass spectrometry analysis. CCAP is mainly expressed in the central nervous system and/or in the intestine in most of the determined insects, such as *D. melanogaster* and *R. prolixus* (Lee et al., 2011; Shi et al., 2019). In *R. prolixus*, CCAP and its receptor have also been detected to be present in salivary glands (Lee et al., 2011; Lee and Lange, 2011; Lee et al., 2013). However, salivary gland-specific expression of CCAP in *P. lewisi* has not been reported in other insects to date. CCAP plays a crucial role in numerous biological and physiological processes in insects, mainly including the regulation of heart contraction, ecdysis, and feeding (Sakai et al., 2006; Estevez-Lao et al., 2013; Lee et al., 2013; Marco et al., 2018; Shi et al., 2019; Shen et al., 2021; Verbakel et al., 2021; Shi et al., 2022a), but its potential physiological function in the salivary glands of *R. prolixus* is still unknown. In *P. lewisi*, three putative CCAP receptors were identified, which were highly identical to their homologs (>90% identities) and highly expressed in heads, indicating that salivary gland-derived CCAP of *P. lewisi* could be considered as an endogenous endocrine factor. It is worth mentioning that the presence of the atypical CCAP in *P. lewisi* venom as a toxin cannot be ruled out given an intriguing example of a CCAP-related peptide discovered in the venom of *Conus villepinii* and having the activity of decreasing the heart frequency in *Drosophila* larvae (Möller et al., 2010).

The insect neuropeptidergic system has been considered as an ideal target for the development of greener pest control strategies. Comparative genomics and transcriptomics provide useful information for appropriate design strategies to develop target-specific insecticidal molecules that could successfully control pests while protecting beneficial species. To date, insecticidal activity and biosafety have been demonstrated for a few insect neuropeptides and their analogues, such as kinins, proctolin, CAPA, and TKs (Shi et al., 2022b). For example, insect kinins and their analogues were determined to exhibit high efficacy against aphids, however, they showed safety to an aphid predator, the common green lacewing *Chrysoperla carnea*, based on the transcriptome analysis information that insect kinins were not found in *C. carnea* (Shi et al., 2022b). Based on the comparative analysis of gene sets of known neuropeptides and their receptors between *P. lewisi* and other heteropterans, *P. lewisi* harbors almost all kinds of neuropeptide signaling system identified in other heteropteran species. Because of the conservation of this signaling system, it seems difficult to develop green pest control strategies based on neuropeptide systems putatively lost in benefical predatory bugs. Although sequence alignment of mature peptides between *P. lewisi* and its close herbivorous bug species showed several neuropeptides like OKB, CCAP, and PTTH, which are highly diverse in bugs, these neuropeptide signaling classes could be potential targets for the development of highly selective insecticidal agents needs to be further determined.

## 5 Conclusion

In the present study, a total of 59 neuropeptide precursors and 58 potential neuropeptide receptor genes were identified through transcriptomic analysis. The present study also revealed a set of neuropeptides and their receptors that were enriched in the salivary glands of *P. lewisi*, providing basic information for in-depth study on repurposing neuropeptides and their receptors into insecticides and targets.

## Data Availability

The datasets presented in this study can be found in online repositories. The names of the repository/repositories and accession number(s) can be found below: https://www.ncbi.nlm.nih.gov/, SRR20681617∼SRR20681631.
