# Supplementary material for: Transcriptome analysis reveals salivary gland-specific neuropeptide signaling genes in the predatory stink bug, Picromerus lewisi
Source: Front Physiol. 2023 Sep 29;14:1270751. doi: 10.3389/fphys.2023.1270751 (PMC10570428; doi:10.3389/fphys.2023.1270751)
Supplement: Supplementary file 1 [file DataSheet1.docx]

Supplementary Material

# Supplementary Data

# 1.1 Supplementary Data 1. Predicted amino acid sequences and putative mature peptides of neuropeptides in *P. lewisi*. Predicted signal peptides, amidation signals, cleavage signals, mature bioactive peptides and cysteines are indicated with different color shades.

**>**SRR10134979.1726511.2 Adipokinetic hormone (AKH) [*Picromerus lewisi*]

MFKVVVILSFILGLTTLCLCQLNFSPGWGKRSQEECKLNTEAVFYIYKT

>Pl_Cluster-8032.0 Corazonin (Crz) [*Picromerus lewisi*]

MWLRLRSLLLVALLVGSALAQTFQYSRGWTNGKRSFPGSQTSCQIQRLRAMLQGKPIPPSYHLLCDLYRLPEDEMKLDQLDKTHVGADDVIEK*

>Pl_Cluster-9760.3123 AKH/Corazonin-relate_peptide_(ACP) [*Picromerus lewisi*] / Partial

…ATSYAVIFVTFILFQEISNSYAQVTFSRDWNAGKRAVSDCSQMSIKSAAAICQMLLGELKSLATCEMRSLLSSRVSEEVDNAPDEFSSRTG

>Pl_Cluster-9760.7812 Agatoxin-like 1 (ALP1) [*Picromerus lewisi*] / Partial

…RPQLILLLCLAAAVSAASPHYFDDDSNNLGEYGTGAFDDYSENDLENIFEAAQKILSETGSYEARGKKCQKSMFGYDLGKRRGCVRRGGNCDHRPSDCCQNSACRCNLWGANCRCQRKGLFQKWGK*

>Pl_Cluster-9760.22773 Agatoxin-like 2 (ALP2) [*Picromerus lewisi*] / Partial

…LALTALLAAAAANSQFESSLDDNYDIGTEQAIYGPQIKRGCIARGGNCDHSSADCCAGTACRCNLWGANCRCQRKGIFTKFG*

>Pl_Cluster-14009.0 Arginine-vasopressin-like peptide (AVLP) [*Picromerus lewisi*] / Partial

…ALLLLTAISLAYCCLITNCPRGGKRSLRPLQRDPDCRRCGPNMEGRCVGRTVCCGPRIGCLVGTPAVLGQCVAEPQLPQLRGGQRCSAGVCLADGVCCSAEGCKMDMSCELEQSVDVCSLPEQQVY*

>Pl_Cluster-9760.4462 Allatostatin/FGLamide Allatostatin (AstA) [*Picromerus lewisi*]

MMLAILISFCLAQICLADLGKDSQPKRLYDFGLGKRAAYTYLSEYKRLPIYNFGLGKRSDDSYYYGKRFNDMDLEYEDDDIKDDYKRMKQYSFGLGKRLPLKTYNFGLGKRSQFDDREASFIEGFDDDKRSNNGHRFSFGLGKRDGAKQEIPGRRSMQYNFGLGKRSQSKDEINPTFNL*

>Pl_Cluster-9760.5343 Allatostatin B/Myoinhibitory peptide (AstB) [*Picromerus lewisi*]

MKLISVIFMVGLAALTFGEELQEDSISPIQQLEDTITMENEKRAWKDLSKPWGKRAWGDLQTGWGKRHVDNDHAVDWSKNDNKKRAWSNLHSAGWGKRAGSDLESVGWGKRGWSDLQSAGWGKRGWSDLQSAGWGKRGWSDLQSAGWGKRGWSDLQSAGWGKRGWSDMQSSGWGKRAWSDLQSSGWGKRSEQEGDEDNEVQKKSWDSLHGGWGKRAADWGSFRGSWGKRDPAWQNLKGLWGKRSFPHSESFEPGLNNLEEEIRRTL*

>Pl_Cluster-9760.5143 Allatostatin CC (AstCC) [*Picromerus lewisi*]

MNFLRRSVVRWALLVTICLIIPPPTAAAPKFTDYKEMGVQYDEYPVVVPKRAAMLLDRIMVALQKAVDEDTVKSAKTRIPIEDTMDLQRRGQQKGGRIYWRCYFNAVTCF*

>Pl_Cluster-9760.17188 Allatostatin CCC (AstCCC) [*Picromerus lewisi*]

MRGLAQAIAALLCLLLVKSLAAQPADKERLMNELDKLVDDDGSIETALMNYLFAKQVVNRLRSQVDVSDLQRKRSYWKQCAFNAVSCFGK

>Pl_Cluster-9760.5017 Allatotropin (AT) [*Picromerus lewisi*]

MKSLPIVILVLCGAVCLARPPQTRPRATRGFKNVALSTARGFGKRDGLPLQQPRLKAEWLANELTNNPQLAGMFVRRFLDVDADGFISPNELLATPH*

>Pl_Cluster-9760.2329 Bursicon alpha (Burα) [*Picromerus lewisi*]

MRIIAFLAQLLVVAYCQSDDTKRPADECQVTPVIHVLQYPGCVPKPIPSFACTGRCSSYIQVSGSKIWQMERSCMCCQESGEREASVTLFCPKAKPGEKKFRKVMTKAPLECMCRPCTSVEESAVIPQEIAGLTDEGPLNQHFVKPQ

>Pl_Cluster-9760.1848 Bursicon beta (Burβ) [*Picromerus lewisi*]

MEWYNVALILLAVSSVAADNEEACETLPSEIHITKEEFDELGRLQRTCSGDIAVNKCEGACNSQVQPSVITPTGFLKECYCCRESFLRERTVRLEHCYDPDGVRLSHNMGVMDVRLREPADCKCYKCSDYTR

>Pl_Cluster-9760.3439 Capability/Cardio acceleratory peptide 2b (CAPA) [*Picromerus lewisi*]

MSNLFFLIASLVLLASCSRAAPGQKTISQACTREKRDAGLFPFPRVGRTFPFTWSFIPLVDPESGERQNKREQLIPFPRIGKSGPKRNGASGNGGLWFGPRLGRLSKRMELIPVAYRQENNVPENLLKSMMKNPSMKGVGNSNDIEDYIDSKQ

>Pl_Cluster-9760.28056 *Carausius* neuropeptide-like precursor (CNP) [*Picromerus lewisi*] / The active peptides are not clear/

MSSERFKLPLVTMWLLLVWCGHMVAASRHPNLIPDHAEVTVQSSVPTEIDNSISPFPYGSGTYHFVSTSFPEIDSRGFHEGMFDGIGDYYPAWSAMYSKRMIRSSDNEEKGGVRRKREVAKEENKPITVEKKAGGRYVRGMEMGSSGFHGDVFNSGFGDFVTMRRRRRLMDKRRPEMDSMGFHGDTFGGGFGEFETMRKRAKILAGIKNYLKRRPEMDSMGFHSDTFGSGFGEFDTMKKRRPEMDSMGFHGDTFSRGFGDFDTMKKRRPEMDSMGFHGDTFGGGFGEFDTMKKRRPEMDSMGFHGDTFGGGFGEFDTMKKRRPEMDSMGFHGDTFGGGFGEFDTMKKRRPEMDSMGFHGDTFGGGFGEFDTMKKRRPEMDQMGFHGDTFGGGFGEFETMKRRPEMDSMGFHGDTFSRGFGDFDTMKRDQQHHLDKKSENV*

>Pl_Cluster-9760.3234 CCHamide 1 (CCHa1) [*Picromerus lewisi*]

MHHLYTLLVMASIIASAPTYASGKTRKLMTPLFVESRRALKPTLQNLFISRSCLSYGHSCWGAHGKRSGDLEPAQFIVPKANSMSDKQARLTEYLLSLSQLQQQKNPEDTAEDVGREEVILMEEPLRLYQILDQLPRSYEGKSSINN

>Pl_Cluster-9760.15552 CCHamide 2 (CCHa2) [*Picromerus lewisi*]

MKAPRHLTVFLILVIVVIQAIAFPYKGERDGESSFRKKPLRRGGCSSFGHSCFGGHGKRSEDYLSQIRRTQKVSPSADIIRQLLNAYHSSSHPLLD*

>Pl_Cluster-9760.10954 CNMamide (CNMa) [*Picromerus lewisi*]

MPPALDKLLQAIQHLINEGSYGRLLLIAVAMAVLSGATSAPLQEPYLQDALAYINTIHSQEMSNGKLRELYEALLRRSGEDIEDDALLQAKIIEYLQNHHLQPKADKRASYMTLCHFKICNMGRKRTLWPRN*

>Pl_Cluster-9760.1237 Crustacean Cardioactive peptide (CCAP) [*Picromerus lewisi*]

MLLYITVLLSVICLAMADEVILQKSMYSPEETTNPIVHRVKRSFCNSFTGCRTWYLPSTDLLGR*

>Pl_Cluster-9760.3778 Diuretic hormone 31/Calcitonin-like Diuretic Hormone (DH31) [*Picromerus lewisi*]

MVSNVLLVTFSLTLGTLILLSTAAESRMYGNNGHRDYFGDLDSEPDPEVILEMLANLGQTITLRANDLENYYKGYLQSSKRGLDLGLSRGFSGSQAAKHLMGLAAANYAGGPGRRRRQV*

>Pl_Cluster-9760.2693 Diuretic Hormone 44/CRF-like Diuretic Hormone (DH44) [*Picromerus lewisi*]

MWVKVLLAWLLAALALVAASSDWSQSSSSHLYSPLLQDEDWRGKRTGPSLSVANPIEVLRNRLLLEIARRRAQKQGGQVEQNRNFLNNIGKRHTMPYRSFDRRFLDSSDYRLSSGEWEAP*

>Pl_Cluster-9760.22683 Ecdysis triggering hormone (ETH) [*Picromerus lewisi*]

MIYLIRFILLLVGALLILTESTCGSIMDDSSEDFIPVMVRRNDFFLKAAKSVPRIGRRNDFFFAKNLKSVPRIGRRNDFFLKAAKSVPRIGRRDEITSIEKEASVAWPWFRDHDFIPRVMKKDLGTDEDISADEPSKEKEEPRLQAIV*

>Pl_Cluster-9760.2139 Eclosion hormone 1 (EH1) [*Picromerus lewisi*]

MDSSKFLAVLLLSCLFAELVPANQVGVCIRNCAQCKKMFGAYFEGQLCADTCLKFKGKLIPDCEDVASIGPFLNRID*

>Pl_Cluster-4557.0 Eclosion hormone 2 (EH2) [*Picromerus lewisi*]

MLAPLVLSLLIGLVSTSQVSVCIMNCGQCKMMLGPYFKGPACAHSCLATFGMTSPDCNNPYSVKAYLKRMI*

>Pl_Cluster-9760.2240 Elevenin (Ele) [*Picromerus lewisi*]

MFCSERRGISSVCLCLFLLSLLVALAACQRSRSVNCKKMVFAPICRGVAAKRAGKILYPNQLQQTYSRRNTNQFMSPEKQLLPYWFYTAKNYQ*

>Pl_Cluster-9760.41598 FMRFamide (FMRFa) [*Picromerus lewisi*]

MLGSMLVGAWLLIHHQCCALGEEEARLRNPLLVDTLTRRSSLEKNFMRFGRSSTGEEGRCQPSPLDLNEIEEQLNNEKRARNPLSSSAKANFIRLGRAKDNFMRFGRGNENFMRFGKSKDNFMRFGRGKDNFMRFGRSERPVQRSGRGRSRDLEDGFIRFGRSLPSPFHPGFIIGPELTLLPPLVLKQRNDNSADNTIRLG*

>Pl_Cluster-4450.0 Glycoprotein hormone alpha 2 (GPA2) [*Picromerus lewisi*]

MAHKFLWCLLLASSFPGLLSKQDAWGKPGCHRVGHTRKVGIPDCVEFEVTTNACRGYCESWAVPSPKETLQSNPRQAITSVGQCCNIMETEDVEVRVLCLNGMKDLVFKSAKSCSCYHCKKD

>Pl_Cluster-9760.25885 IDLSRF-like (IDLSRF) [*Picromerus lewisi*]

MSPAQTALVLAATVGVVCIAWPHHVMAIDLSRLYGHVSTKRNSEACHPYEPFKCPGDGACISIQYLCDGAPDCLDGYDEDSRLCTAGIVDVHFLHEISSDAAKRPPVEETSSFLQSLIASHGPNYLEKLFGSKARDALAPLGGVEKVAITLSESQTIEDFGAALHLMRSDLEHLRSVFMAVENGDLGMLKSLGIKDSELGDVKFFLEKLVNTGFLD*

>Pl_Cluster-9760.20378 Insulin-like peptide 1 (ILP1) [*Picromerus lewisi*]

MIKQLLLVSLFITVFAQWDVFTENDAKRSQIYCGEELNKALYLICGGLYNSPYKKSLESYDDYYSDYSLLDSLEQSGADFRFPFHPKTRAMSALQYKRLKRGVHDECCRKSCSINEIKGYCLKKN*

>Pl_Cluster-9760.11382 Insulin-like peptide 2 (ILP2) [*Picromerus lewisi*]

MFLWTPRILLFISLVVICSARQFCGSDLADVMSLVCSGRGYNVAFKTEYSPAEKRFKRGIVDECCRRGCTWSTLETYCSPESSDLQIRKRSDFTNKWQPDTADTEEKKTDKFKYDTNEIKIPQGIENQQKWRFNILVENTKPGYPSRYYSLLEGLNRQVQIPRGPSVVNKVKRNDIEEGKGSRVQKNPNALPDALNWPNFLIDGMKLKHEKSEEEKIQESSTASVPIRHRHRKSDLSRKIIKDNGMNTSQIGTVPPYFQGRTLVLPPSRKL*

>Pl_Cluster-9760.23228 Ion Transport peptide (ITP) [*Picromerus lewisi*]

MHQERRALAGLVVASTLLSCLVAVPTSRSIMGHPLNKRSFFDLQCKGVYDKSIFARLDRICEDCYNLFREPQLHSLCRKNCFTTDYFKGCLDVLLLQDEMENIQTWIKQLHGAEPEV*

>Pl_Cluster-9760.16172 ITG-like (ITG) [*Picromerus lewisi*]

MLPAVLLLSAVLHPALGWGGLFNRFSPEMLSNMGYGGHGTSLRGQSYLQVENPGAMETLQEMQEGEMEGPCYGKRCTANEHCCSGYVCIVVDGTSGSCMFPYGLGQGELCRRHSDCDTGLVCSDTGEGKTCQPPFSAPKHYSEDCTLSSECDIQRGLCCQFQRRHRQVARKMCSYFTDPMVCIGPVASDLVKVQVERTAGEKRITGKTPIFL*

>Pl_Cluster-9760.3008 Leucokinin (LK) [*Picromerus lewisi*]

MGVLWLLSMTIICHTTQRTSAWTGGNQSSTSTSKPVFENHTINVLEELPLSKSIVNSSEENMREKRGTMSDENSIEPDLEKRDPKFKFSSWGGKRNHIPEEKRAKFSSWGGKRSYDLGSNEIDDDIEEEKRARFSSWGGKRSDYDADKRAKFSSWAGKRDTNEITENKRSKFNSWAGKRMDLEGDKRAKFSSWAGKRARFNSWAGKRSDGTFYDEKRVPFNSWAGKRSDLEPENEYEPAEDKRAKFSSWAGKRSYLDPDGDEEETNEEKRAKFSSWAGKRAPDYVGIAWNAPDNKLRYYSKAGGAQSSENIMKLWQDLMSRKRARFSSWAGKRSDETMVDSENLVRYLEPEAEEKRAKFSSWAGKRDDTDANKLEGWEGNQGNESVGDVNNEREYPSMIVKRSAYSWNSPSVAYKRKPIFSSWGGKRSDDSHTIQARSLPMQRVLLNGVKDRSWGSLLRPIRRGPDFYAWGGKRST*

>Pl_Cluster-9760.8672 Myosuppressin (MS) [*Picromerus lewisi*]

MNFSWMWTVLSAGLLACTMAAPGPDCSPAVLQELPARVRNMCAALYQFSNALQQYIEENPSYQPVAREASPIYESGVKRQDLDHVFLRFGRRR*

>Pl_Cluster-9760.1632 Natalisin (NTL) [*Picromerus lewisi*] / Partial

…FVLIVLLTLQLQRICCEERRTDVMRAVLSGPAEPGFWPSRGRRSDSSSEEEPPPFWAHRGRGLSSYLLLHPEEPLWLTARRKRSQDH*

>Pl_Cluster-9760.2157 Neuroparsin A1 (NPA1) [*Picromerus lewisi*]

MVSSLLVLASFAWASCASRMSCRGIDCERKPENCSHGPVEIRKRWVCGKGPGESCGGYMEMKGKCGEGMYCDNCGLCRGCSQRVLILHKRSECNNTTCTRRPSRFV*

>Pl_Cluster-9760.3086 Neuroparsin A2 (NPA2) [*Picromerus lewisi*]

MCPQAFVVFLFVVALALAAEESLFFPCRGEDCDLMPDGCLYGTVRDACGRVVCAAGPGERCGGRENHLGKCGEGMSCKCGKCRGCSIARLMSGIIECEYSNPICS*

>Pl_Cluster-9760.2919 Neuroparsin A3 (NPA3) [*Picromerus lewisi*]

MYSQIFLLTSLACLVLANAHNFYQCEPCRGEECNVQPEGCLFGISRDPCGRLQCMSGPGERCGGRDNHLGKCGDGMNCKCGKCRGCSIDRLKVGIVHCDPNTTPVCY*

>Pl_Cluster-9760.3188 Neuroparsin A4 (NPA4) [*Picromerus lewisi*]

MYSFTFLITAMVCLSLSNSFEFYECQPCRGEECNLEPEGCKYGISRDACGKMQCMAGPGERCGGRDNHLGKCGDGMTCKCGKCRGCSIDMLKVGIIECDANTTPVCY*

>Pl_Cluster-9760.3111 Neuroparsin A5 (NPA5) [*Picromerus lewisi*]

MNSVVFFLSALAFVVLANARNFHQCQPCKGEECNVQPEDCKYGMAKNICGRWECMVGPGQRCGGRDNHLGKCGNGMTCVCNKCRGCSSDMYQKGIYECTKTSSPICY*

>Pl_Cluster-9760.3160 Neuroparsin A6 (NPA6) [*Picromerus lewisi*]

MYSLVILSALLFVVFAHSRDYHPCQPCKGEECYVQPEGCLYGIARDACGRLQCSSGPGQRCGGRDNHLGKCGDGMACMCGKCRGCSIERLKAGIIECDANTTPICY*

>Pl_Cluster-9760.2950 Neuroparsin A7 (NPA7) [*Picromerus lewisi*]

MYSLLFLFTAVTTAMAFSFDYYPCKPCKGEECYLEPEGCKYGITRDPCGKMQCMAGPGQRCGGRDNHLGKCGDGMTCKCGKCRGCSNDRILLGIIECDPNTTPLCT*

>Pl_Cluster-9760.1926 Neuropeptide F (NPF) [*Picromerus lewisi*]

MRTWMVCSSICLMVMACQADPLPADAMARPARPKSFASPDDLRTYLNQLGQYYAVAGRPRFGKRTSMHSPRLHIPTDGMNYRYPAVPDASELYDFLYQPLAE*

>Pl_Cluster-9760.21862 Neuropeptide-like precursor 1 (NPLP) [*Picromerus lewisi*]

MTTTLCALALAVFLVQCVLLCLVNPKETLAFSPRSESLAVLPSGASQVRGDVEKRSITAVRSDQPPEKRYIAALAKNGDYPRLAWNKKYHSLEDTPFGQDPIIEDTKRYLGALARTGDLRVAREREDKRDDVDSLIRDIASADDLRRLRLEALREELLKERDDEPEQDDDTDDKRSLASIARAGGIPGKRSVEALARIGLLKPITTTHDFTEDINDYEKKISSDEFEDEKRGGVSSIARNGYYNNKRTVDEELEELMNEVYGIGEKRNVASLARGFNLPQGGKRSEEVEDKRNLQSVMRDRGGKRDGYTNNAIPFVNNDKRNVGALAKNRDFPYAYRFGKREVSEVENDEMSKRYVATLLRDGRLPIGPDATPEHETSNLKDDNSATEGKESTSQIAEDKNQSVRRKKDISEVEKEENHTRSKRETFIAPIAGEVAVPPSGDLHFGGMLSDNRWADESSPLNKRFFGVDTWINPEKRHIGALARGGWLPRAYLRSGRQPSGEGRWQDAWWSTS*

>Pl_Cluster-9760.15366 NVP-like (NVP) [*Picromerus lewisi*]

MGMILKLLVQIFCLSVVAYAIPVSVLEDIKDAQLQSPLRANKVKRAQEVLMFGNQQNRAVNTYGNPKQEKRDVGDNSLPDDVPTLPNIIPQMEEQSYDTNQLGVGEKYEKSYPYISREPNYGMLLRNAALKESLHNEPVLGYGDLSLYNMMDARRKRDVHKGGTPTKNSFRSKRDYDPYDLSPEEIISLMRLLETQRRQSGRVHDQRNNWPGYGTDSEDFDLPERHEENEVDSDSNFSHNGVWMDAPISSLNQNPHDSDFWPRKFKRFMVSKRRSDEPLSQLNSPYDFPEGVPLQRRFIL*

>Pl_Cluster-9760.25576 Orcokinin A (OKA) [*Picromerus lewisi*] / Partial

…LCISCCYVSFAGGGNLLRDLQSGLGRPRGYMIPRQNREYLDSISGTTFGSSKRFSGPLFAADKRNGMDEIDRVGFNTFIKKKNFDEIDRSGFDGFVKRNFDEIDRAGFNGFVKRSSE*

>Pl_Cluster-9760.28609 Orcokinin B (OKB) [*Picromerus lewisi*]

MSPIVFCLAVIVCVSAAQPLRSEMATEELYPNYAYEEAKQRNLDSIEPAVTNKELMTQLDRSAWMRRGTLDTIGGGHLVRNVETDLRNNYGYYPDGHLDTLGGGHLIRNLDTIGGGHLVRNLDPIGGGHLLRNLDPIGGENLVRNVDTTGGERLDGNLDSIGGGHLIGRYRRELDPIGGPNLVRIYEGEDLAGNLDLLDEKNSGSHGDAQVGGSFVRNADTTQGSRYRRQVDSLGGPNFVRNVETAEELRLDRNL

>Pl_Cluster-9760.11083 PaOGS36577 [*Picromerus lewisi*]

MWALLATTCLLAASAAADDSLRSALHAVQKRQRDLTPQEPLYFNRPNMMYSEGQPEDIGYGYQKTVPGTPGLFEALPIPASALGASYDSYKPLSGFFVDDKYNVPSSKRSIFRERGDSADALQRIYEENQQEDDGPLLIPSPFRERMSEKSRLQNANALVRNFGGLNRQPEDQLGDDDYINVLNNIWEKYKNEEDPEDITEADVEDILEYLARKEEKKRQQYGNYDTGYDFFNAPMSWTKRDPRMDGHHKRYHETLFDERYPYRGPQKRYPITKRSPTVVASTSVSHRHKKNTPEKQQTDPKVAAELNNIFSSPQKGSKNHSEVTTTTQVPNTTQTPGQKEQTGDNSSLKPLDVKKKSINWSDYFGIDRRKKSADSEINNEWLARKYLEKYGYFDKDKDQMMESQKKRPDDVDNKMRAMEDLIVDQAIKYTGAHEGTTDSKEMQEVKDKVMAQLAAAYSLEKMRRALGEFKASIAAQRVNNPSGNGMAIPKQEQDFKQKMVKDKSEVASGKMEKKDIPEIRNEVAEIGNLHGRECPVLQGVEMRCKQVASMAGDRAEIFLPLCNLHHMCYLCGAVLRAASPRECDTAFLEEAESVCREEPSCYYMARRTLSVARSLKADPNLVCDWRNSPCLAQFLSLTTEK

>Pl_Cluster-9760.21295 Parathyroid hormone (PTH) [*Picromerus lewisi*] / Partial

…RLIILLLAAAIAHGYRFRQKRVSDQRLAELETLVALSNMKGNIKTIPYGYGSVDPYKAGRKRRSSGALLDRLIESIAHEDDNSIGQERPVIQWPPYFWAANDQPE*

>Pl_Cluster-9760.2504 Pigment dispersing factor (PDF) [*Picromerus lewisi*]

MKIMISIIFGALVCQAMFTDVSSLPAYTLNDKVLDKLLLQTLSGKETPIWLSELLKSEDHTHKRNSEIINSLLGIPKVLNDAGR*

>Pl_Cluster-9760.7942 Proctolin (Pro) [*Picromerus lewisi*]

MYRQSIACFLALMMLLALTEARYLPTRSQDDRLLRLRQLLKDLLENDMDPVEHPISGFEPRLYKREAIGYDRPVQYLH*

>Pl_Cluster-7513.0 Prothoracicotropic hormone (PTTH) [*Picromerus lewisi*]

MFLYLFHDRVLGAIILPLFALNALTNLGKACTVNEECAGRPKLLDLGRGYYPQFLFTLECDSKCLPYICTPQKYNVKVLRRRRIEDITVDGSLPESLRESWRFVYKPVTVACLCNTYNQEQ*

>Pl_Cluster-9760.41631 Pyrokinin (PK) [*Picromerus lewisi*]

MAPHCCWFPFCLLLITSFISSYGAEEDRVQRTEEDDDLETAIVGIAPWAAFLPISGGERGSRQLVSFRPRLGREEEVLAESSRSPPFAPRLGRFYAPFSPRLGRRRS*

>Pl_Cluster-9760.4458 RFLamide (RFLa) [*Picromerus lewisi*]

MHILVVVLCCSLTFGITFPQEIDETMDISNSIPTDLGEAEEISDPDLLSEYLSSLLVDETAPLPLIYLPEKRSRYYRRYPWKRQNGRNYEPDSYLCTPSRQDVFHLLMALHDARSGNQDRTVHFCNRRRPARAIFTNIRFLGRR*

>Pl_Cluster-9760.2102 RYamide (RYa) [*Picromerus lewisi*]

MWWSYLFVCILLFSTVPSYSTPLGFYGSSRYGKRTAITRREPRSGIFWTGSRYNRRGSDILGMKSTRKGSDNFFMGSRYGKRTGVDENKTTFDLDLCKFNKYLYSCKSSSR*

>Pl_Cluster-9760.7168 short Neuropeptide F (sNPF) [*Picromerus lewisi*]

MKFALPVMGFFVMVLLPAISSAPASPDYESVRELYEVLMGRDPQVADLWAGHRLVRKNSNRSPQLRLRFGRRSDPAFMQLGDHGMEHSMFDSLDN*

>Pl_Cluster-9760.41261 SIFamide (SIFa) [*Picromerus lewisi*]

MSRALFLSFLVIAFAMLVFDVASASYKKPPFNGSIFGKRSGPPTDYETAGKALSTMCEIASEACAAWFPVQENNLN*

>SRA:SRR10134979.954898.1 Sulfakinins (SK) [*Picromerus lewisi*] / Partial

…ADCRSYRGREVEDETDLELAKRHFNDYGHMRFGKRGGVTEDKFEDYGYMRFGR

>Pl_Cluster-9760.11446 Tachykinins (TK) [*Picromerus lewisi*]

MRITTLVWAAVAVTVLANVATAQEVRRAPSMGFMGMRGKKDMFPDDDNSIEEYKRAPLMGFQGVRGKKAPSMGFMGMRGKKDDGQDLWDEEKRAPSSGFFGMRGKKAPAAGFFGMRGKKGPSSGFFGMRGKKGPSGFLGMRGKKESIDDIDSLLQYLRDSEARQDVEDMMETRAKRFVDDSFQEPQSEQDFL*

# 1.2 Supplementary Data 2. Predicted amino acid sequences of neuropeptide receptors in *P. lewisi*.

>Picle_ACPR / Partial

CVSVDRYFAILHPLRVNDARRRGKIMLLIAWIFSFICALPQSMVFHVSEHPHYPGFKQCVSFDVFEPALETAYNLFCVSAMYFIPLIVIIFAYTCIMWEISKKSRETRADETDNDRVRGRMRLRRSDMSNIERARSRTLRMTITIVAAFIWCWTPYVVMTLWYMFDRETATKVDSRIQDALFLMAVSNSCMNPLVYGSYAMNFRRECQTCFCYLFNSNQQLQRRSYRCRWWQDCGRSLCAAVCDYTGQR*

>Picle_AKHR / Full-length

MEDGPEYRRVWFPDSPVHVTEDNKTFLIPIDMRFNEGHKVSLVFYSILMVISAVGNISVLAILLKTMKTSRSRINMMLIHLAIADLLVTFILMPMEIVWAATVSWWFGDLACRAAAFFRTFGLYQSCFVLVSIGLDRYYAVLNPMKLSDANRRGKIIIKCAWMMSALCSLPQTIIFHVEKHPNVTLYEQCITINSFSSKAQEFAYSFFGMLSMYLVPLILITFFYGSIFIEICKRSKDHQANSDKLRRNNLAFFGKAKTRTLKMTITIISAFFICWTPYYIMAFWYWVDKDSATQVDQRIQKALFLFACTNSSINPIVYGAFNIRKRKNVSRRTRGSNTCTTEIKLQSAGQKNSLQ

>Picle_AstAR / Partial

…APPFSGPGFPALRYVRCANLTDNLTEVSFCGNVSYQILGEEEPEVMVMENIVSIVVPILFGLIVVLGLFGNALVVIVVAANQQMRSTTNLLIINLAVADLLFIIFCVPFTATDYILPFWPFGDVWCKMVQYLIVVTAYASVYTLVLMSLDRFLAVVHPIASMSVRTEKNAITAILVTWIVIVVASIPVFLCHGEVNYTYSSMEHTACVFLDRDTHVPNGFNKLAFQVSFFTTSYVIPLALICGLYLVMLVRLWGGTAPGGRCSAESRRGKRRVTRMVLVVVAIFAICWCPIQVILVLKSMDRYEITNTSIMIQIVSHVLAYMNSCVNPILYAFLSENFRKAFRKVIYCGPEGAPHLHHVNGRNDAEKSAVSTKTTRSTNII*

>Picle_AstBR / Full-length

MKDMEEKYTILEGNATIYNLSSAENATDRNISVEYLNVTNEVPIQFAEPMYGYIMPFLLLITIIANTLIVVVLSKRHMRTPTNAVLMAMALSDMFTLLFPAPWLFYMYTFGNHYKPLSPVGGACYAWSIMNEVVPALFHTASIWLTLALAVQRYIYVCHAPVARTWCTMPRVLKCIAWISFIATLHQSTRFIDRDYLPVNISWGNQENVTVCRVQIAHWVEKGISPDVYFIIYYLFRVVFVHAVPCISLVALNLLLFRALRKAQEKRDKLFKENRKSECKRLRDSNCTTLMLIVVVTVFLVTEIPLAVVTVLHVISSSVTEILDYSVANALVLFTNFFIIVSYPINFAIYCGMSRQFRETFKELFIRGAVQVTRRNGGGSSKYSLVNGPRTSTNETVL*

>Picle_AstCR / Full-length

MAAEEGSGWLWSENGTNSSQGNDTQYCGNLDQPTLYIFTQVLYAFVCIVGLLGNSLVIYVVLRFSKMQTVTNMYIVNLAVADECFLIGIPFLIATMSLQLWPFGNIMCKIYMTTTSINQFTSSIFLTIMSADRYVAVCHPISAPKMRTPFISKIVSLSAWTASAILMIPVFMYANIMDSEHVNSCNILWPESENLSGQTAFTLYSFILGFAIPLVLIFCFYFLVIRKLQTVGPKNKSKEKKKSHRKVTKLVLTVITVYVLCWLPYWITQMALIFTPPKQCQSRITVTVFLLAGCFSYSNSAMNPILYAFLSDNFKKSFLKACTCAAGKEVNATLHLENSVFPRRTARGGSERAPARRSEPQEAGPLVSRTEASTTALTSRSNITITSDSVTPVKNGVKVSVTPTQL*

>Picle_ATR / Partial

HISSSLAPLRLLIKKMNKSQVLNETYEKCTNDYCISDDDYWGLIQSHIMPKHYEWLLIVLHGIVFLVGLVGNALVCLAVYRNTGMRTVTNFFIVNLAAADFMVILACLPPTVIWDVTETWFLGKTLCKLILYLQTVSVTVSVLTLTFISVDRWYAICFPLKFISTTFRAVSAIIVIWVIALVLDLPEMMTLTTFRREDLRIETILFTQCEASWDQSFEQTFSAVKIGFLYVLPLLFMSVAYFQIVKVLWSSDNIPGQIVGGESCRRGGTF

>Picle_BurR / Partial

IFHCTVIGMLAVVDASTLGEFEMYAIPWQMSAGCQLAGFLGVLSSELSVYTLAVITLERNYAITHAMHLNKRLSLKHAGYIMLCGWTFAIIMAILPLIGVSDYRKFATCLPFETNTSWSLTYVVFLIFINGVAFLILMGCYLKMYCAIRGSQAWNSNDSRIAKRMALLVFTDFLCWSPIAFFSMTAAFGWQLVTLEQAKVFTVFVLPLNSCCNPFLYAILTKQFKKDCVLICKAIEESRVTRGIGRCRHSSNFSNRQTPANTNSLVDRSSRDNQVHQPCNCNVKLLGDSVSSKRTPKGWLINKVHWMRSCLGRNSNTRHRTRSDQYAYQIAEIQQKQHKRASSVSSSENFSSSRSDSWRQNHHHCGLPMRLLDPKRRASSWIITRKTSQDSNLSSSRNDSSGSATTNSTTMSRVSRSSNSGEVRPKPRLTRQSAIQDECDLFGSPAKLTVRFLTTIPSAAETSVQHEDDSPCYAILHASQEQSSNTSQSPSPNSAKHNNSTSKKKQNS*

>Picle_CAPAR / Full-length

MNRNSTYLMDELTRYLNRTKANWTLDDYMELTRGPKHLPISIVLPLTIIYVSIFFTGVIGNTLVCWVIIKHKMMHTATNYYLFSLAVSDLMLLILGLPNDLSVYWQQYPWPFGETFCKIRALTSEMSSYTSVLTIVAFTTERYIAICHPLYSYTMSSLARVLKIILAAWLVSLTCALPFAAYTTVNYIDYPLYSGNIVEESAFCAMLESNIPEWLPIYELSSLLFLIIPIIIIVVLYTKIAMKLRERSDYSLGTRLEGSVHSRKSHSQSRKSIIRMLVAVVSMFFICWAPFHAQRLIYLYGKSLPNYVTINEWMYYITGALYFFSSTVNPILYNLMSIRYRKAFKQTLFGGQNNNKRGQQSSFRESSLGAAEISEDVEKNKCSSSEVKGDSIVRVNNHNVTNGNCVTVRTGFFPKNLLILPPNSQTSKEGGINSLKAVTPSLFLSNQNTAKETYI*

>Picle_CCAPR1 / Full-length

MESTPPSNCWTCNNNETDINSFYFYETEQFAILWLLFILIVFGNSAVLAALQCGNKPKSRMNFFIMQLALADLCVGVLSVLTDIIWRSTVTWNAGNLACKIIRFSQAVVTYSSTYVLVALSIDRYYAIKYPMNFSGSWRRARLLVILAWCVAAVLSIPIGLLYHEKKIQGQLQCWIEFAEAWQWQLYMTLVATAVFVLPALIISICYAFIVFTIWAKGKQYTHKATSEDTRRASSRGIIPQAKVKTVKMTFIIVLVFVLCWSPYIVFDLLQVYGYIPRTQTNIAVATLIQSLAPLNSAANPLIYCLFSASRTNR*

>Picle_CCAPR2 / Full-length

MDWVIRDNYSTISNGSQPEAINSFYFYQTEQLSLLSFLLAAIVLGNVSVLLALLLSKGRKSRMNFFIMHLAFADLSVGIISVSTDIIWRITVAWKAGNVMCKLVRFMQAMVTYSSTYVLVALSLDRLDAITRPMNFSRGWSRARVLVASAWMLSAVFASPILVLYEEKLIQGSLQCWIELGSELKWQFYMCLVALSLFIVPALIIAGCYTIIVFTIWRKSVHLAPHHQAMPLKDGDKHEEIGDLRRASSRGIIPKAKIKTVKMTFVIVFVFIICWSPYIIFDLLQVFGHVPRTQTNIAVATFIQSLAPLNSAANPVIYCLFSTRACTTLGKLPPFSWICCCFVSRGPDTSSIIDTVTSTLRRATMRTQDNL*

>Picle_CCAPR3 / Full-length

MDEEEIALQAGLNETSEINTYDFYATEQLAVLWVLFLLIVLGNSSVLLALAFNKNRKSRMNYFIMQLAIADLAVGLISVLTDIVWRLTIAWHAGNVACKVIRYLQVLVTYGSTYVLVALSIDRYDAIKHPMKFSGSWRRAKLLVAIAWAVSAIFSLPILVLYEEKLVQGQLQCWIELPYQWQWQLYMTVVSVALFFIPAIIITACYTVIVSTIWRKGSTVIVIKRSYKGITTDKVRLEHDHESRRASSRGLIPKAKVKTVKMTFVIVFVFIVCWSPYIVFDLLQVYGYVPKTQTNIAVATFIQSLAPLNSAANPLIYCLFSTRLCRTLRRVPPCSWLNDLFSQCCPGLASATCLTMDHSSTLTSSLQSSRGSRLSSKHVTVL*

>Picle_CCHaR1 / Partial

GDLLVIITCVPFTSTVYTFSSWPYGLFVCKLSEFAKEISIGVSVFTLTALSADRFFAIVDPMRKLYSSIGGKGATGCTIMIAIAIWLLAMFCASPAAIGSYIRVFKKPNSNVTFFETCYPFPEEWGPLYPRSIVMAKFLVYYAIPLTIIGFFYILMAHHLVLSTRNMPGEAQGQARQIRARKKVAKTVLAFVVVFAICFLPHHIFMLWFYNYPKAQVSYNNFWHIFRIVGFCLTYINSCVNPIALYWVSGTFRKYFNKYLLCWRGYSDESRHNRRAWDSSTLAHLHQSTIRRNDTTIQECTVLTTFANGHQGP

>Picle_CCHaR2 / Full-length

MDNCTECDEYVPYAERLETYLVPVLFALIFLVGVLGNGTLVVIFVRHTKMRNIPNTYIMSLALGDLLVIVTSLPFTSTLYTIESWPYGDFVCKLSEATKDISIGVSVFTLTALSAERYCAIVNPIRRHISTKPLTIVMAFVIWIMSFILALPAAIFSNVQEAYLADNRTIEFCSPFPPQYGPPYKQSMVLFKFLAYYACPLCIIAGFYILMARHLVLSTKNMPGEMQGQSNQINARKKVAKMVLAFVIIFFVCFFPYHTFMLWFHFYPTAEQDYNEFWHGYRIVGFCLSFINSCINPIALYCVSKAFRRYFNRYLLCCKRSDTPCNDVTMTHKSSSIKHNRQNSVITSHYTLSNAEKI*

>Picle_CNMaR / Full-length

MILKNTSEGAGLINTVMAGTEDFQNATNGQQNLTVIEHDASERVIGAIMTYSIPVIVSLGSIGNFLSILVFFSTKLRKLSSSYYLSALAVTDTGFLFCTLITYLPTHDIGLFNQEGVCQITTYFAQVFGFLSPWFIVAFTVERFIAVRYPLKRTSMCTVSRAKAVLAALTILAVGACLPFLFLTGIAIKHHNITEEGEERMEVVEVCQLMEGHEKLAETLNHLDTIVTLIIPFLIIVSLNTLISRTVCRVARVRRSMTKSTGTAGRQSAKQRTSSSQTKVTEMLLVVSTVFIVLNLPSYAIRMYVYINQKGEDERAVVVIQQYAQLLFYINFGINFVLYCVSGQNFRRALISLFCPDIRRRAETTQVTTVVSEYTRSASTRRTMTVNGNWREAHEMVPINRAQHHH*

>Picle_CrzR / Full-length

MGKDTYLRELGPGRYALPQELCERFNESGVNLTRIECLEHAPTLTSEAMTRAIVLAVMAVISLIGNLLTIFSIAGTRAGRRRKNQTWSAVYALILHLSISDLFVTVFCIAGEALWSYTVQWRAGNIACKIFKFLEMFSLYLSTFILVLIGLDRFIAVRYPIRAISTAKRCSRFVAIAWVLSIILSIPQLVIFHEGKGPFFEDFYQCVTYGFYTEPWQEQLYTTFSFVCMFMLPLFILITSYVSTIVTISKSDKIFQNESINTVRKYDINRRRLIHKAKVKSFRISLVIVVTFIIWWTPYYTMMIIFMFLNPDKHLSEELQKGIFFFGMSNSLVNPIIYGAFHLWRPKKAGSLRSRGELNKYTSIKRSSTWERRRDYICVTGATVEPQSQLK*

>Picle_ETHR / Partial

MIAAGNESVEAAGNSTYGGGLLFPPYVRAAYILLCVVILGIGVVGNVMVPLVIVKTKDMRNSTNIFLINLSVADLMVLLVCTPTVLVEVNSEPLIWVLGEEMCKAVPFVELTVAHASVLTILAISFERYYAICQPLRAGYVCTKARAMLICLLAWAFAALLTSPIAHITEYSVEKNNGKAGCYAVPDSFWRKFFFYSILTIFFILPLSILIVLYTVIARHLIRDPGTANSGDNIRARKQVVLMLATVVLSFFLCLLPFRVFTLWVIVSPDDITALGEQTYLNLLYFCRLMHYLNSAINPILYNLMSSKFRQGFGRLVGIRRKRHLLLLRHRATFSSTLSRSSTRLPRGSPDLSWRGASLDSRHRNGSIRRSVILRSSLLNHNKQPEPPPQPESYV*

>Picle_FMRFaR / Full-length

MDSVDWSNFDNRSNNSSLLDSSYDDMAIDESELLFEFITNGVLLNLVGILGIMGNIISMVILSRPQMRSSINYLLTGLARCDTVLILTSMFLFGLPALYKYTSTPFLAGYYFRVYPFLSPIVYPLALIAQTVSVYLTLTVTLERFVAVCHPLQARSLCTYGRARLYVVLIIIFSTLYNLSRFWEVTLETEFIPSLNITIYTPVPSELRNNHVYISVYIHWLYLLFIYFLPFSCLAVLNAAIYRQVRKANQERQRLSRLQKKEIGLATMLLCVVFVFVLCNILALVANVLEAFYGIILDRMVKLSNLLVTINSSVNFIIYVIFGEKFKRLFLKLFCSQVPRACYGGRDSPDCATLHEDSVMLSNGEARLRANGSVKRVARALPCVYYPARHQAAKWHHDTTTTTTLNQM*

>Picle_GPAR1 / Partial

…WFWSHSGNYFPGDLVSTYEILLLLFTFLCNISNGRFLQSNISPLIASLNFKDQYPDFSLNYNETISDETFHQVQTDIPKNKIEALCGNLTGRYVSCYPKPDALNPCEDMMSWLWLRVSIWFVISAGIVGNVAVLVVLCLSKTEKSVPRFLMCNLAFADLIMAAYLLMLAIMDMISSETYFNYAYDWQRGYGCKIAGFLTVFASQLSLFTLSLLTIERWFAIRHALYLNMLDLQITSNIMIGGWIYSLVMALLPLIGVSSYSTTSICLPMDSKNYISQMYIITMIVVAGCAFLLMCICYTQIYLSLSYETRHSISEGAIVRKMTILVGTNFLCWAPVAFFSFTALAGYPLITISQSKILLVFIYPINSCANPYLYAILTKQYRKDCISILSR*

>Picle_GPAR2 / Full-length

MCVKKVSVSECTVRRSRTGLHLKVRSGVGWRESAPSVRRRRPDQQHPGSAALGRPAMALAIFLLLLPLHPSKGTNVTELAPFLMLDDQDLSGLCRCWDEPSSPECECRGETMKSVPGNLSHGLERLTIKQSGMTELRNDSLLPYRDTLMELTLLNNQLLSHLDPGLFSNLPNLKTLYIVHAPLLKSLDVSVLNIQLPKMMVMRIVLCGLEELPQITQVEPNQHITMVDMESNQIKRLGRHQFKITADHLVLNHNQIEVVEDYAFEGSQIEKLSLGGNKELTRLDHHAFSGIKSLRVLDLSDTSITYLPTTGLQDLEKFYLENVRTLKVFPSIYNFPHILQVSLTYAYHCCAFHYPSRHDPTRYRKHQELQALMAAKCPKMRGKRSWGAPISRGHHQTSDGFEGTFHQEAFNNISKTRGVKMLAECGNLSKDYRQVDCTPVPDAFNPCEDLMGNWGLRVAVWFVAIAALLGNTAVLLVLLSSRFRMTVPKFLMCNLALADLCMGVYLLMIAVMDAKSIGDYFNYAIDWQSGNGCRIAGFLTVFSSELSILTLTVITCERWYTITYAIHMNKRLKLSTAMKIMAAGWLYAILMAALPLVGVSGYFKTSICLPLEKSATVDIAYLTTLLTFNGIAFWVICLCYGKMYCSIRRGRDAGAALYSSDMTVAKRMALLVFTDFACWAPIAFFGLTALVDYPLINVTNTKILLVFFYPLNSCANPYLYALLTHQYRWDVIILLSRYGLCTERAARLKAGGTSGGGGPGGGAARGQRPAGRGSVLTTLTSLDCARQHSLGPLPEANPPANNGTTHGTSTV*

>Picle_ILPR / Partial

…LIHCFSNEDVFKHYVKYNFRLTDEFRFCCLAKQVPSCWPKRDEFSSCEDLMSNIVLRICVWVLALVATVGNIMVIAWRARYKHCNQVHSFLITNLALGDLLMGSYLLLIAVVDWQYRGVYSIYDTSWRTSKLCSLAGFISTFSSELSVFTLTVITLDRFLVIIFPFRVRRLEMNRTKQLMAFGWIIAIAISAVPLLQIDYFKNFYGRSGVCLALHITPDKPNGWEYSVFVFLFLNLVSFTVIAIGYLWMFLVARTTQHAVNKDRRASESSMAWRMTLLVATDAACWVPIIILGVVSLAGFTVPPQVFAWVAVFILPLNAAVNPVLYTLSTAPFLTPARQGLFSFRRSCKLSMSQDNRRTYMSTINHYNGCGSVELYPMTRRSTRFRNSLDTSLTDHGVVLHMQKLK*

>Picle_LKR1 / Full-length

MNCSNSSIGCAEGGELYEPLYEPPTYLTVFLSICYISISVAAVVGNGLVIWVILTSRRMRNVTNYYIANLALADIAVGLFAIPFEFQAALLQRWVLPNFMCPFCPFVKTLSISVSIFTLSAIALDRYRAILYPLTARASRVHFRVVISVIWIAGGVMAAPFAYGLRVTKAPVPYLPLNSSELIFDYCDNMNMPPELYESYRTTLVLLQYFLPLIVISYAYARVALTLWGATAPGNAQTDRDSNIMRNKKKVIKMLVIVVVLFALCWLPLQTYNVLQNITDINEYKYINIFWFGFDWLAMSNSCYNPFVYAIYNEKFKREFQLRLRAPCIKKKHRGDPLTRELSGFESSRFDWKRASTMKNGMPATLSVSLLKM*

>Picle_LKR2 / Partial

…GGIIVLLSVFYGTISVVAVVGNFLVMWIVATSRRMQNVTNCFIANLALADIVIGLFAIPFQFQAALLQRWNLPHFMCPFCPFVQILSVNVSVFTLTAIAVDRHRAVLNPLSAPPSKLRAKLLLAFIWILAAVLATPMAVALSVTYVDEHDHAGRIYKKPFCNNTRLSNNGMMAYRMILVSVQYLTPLCVISYAYARMALRLWGSRAPGNAQHSRDANLMRNKKKVIKMLVIVVALFAICWLPLQTYNVLQDIFPQINGYRYINIIWFCCDWLAMSNSCYNPFIYGIYNEKFKIEFKQRCPFRQSRRWRQSFGGDSMDLDKTLHRYGSMHQPTRWVRYSSRRRSTFSPSQHYVYHCANSNVVHHHSSHTDIEELCL*

>Picle_MSR / Full-length

MNGTAVRERWCGEAFDSLHDAYKEAHGYSSLVVCLFGSVANVLNIVVLTRREMSSPTNAILTGLAVADLLVMLEYIPYCWREVLAVRPKRDKFSYGHALLVLLHAHFSQVFHTISIWLTITLAVWRYIAVVYPQRNREWCGMQQTIIAITSGYLICPILCIPLYLAFDIQPKRTILDEFGNKPTANTTKFTNTTLYYVDLSELGRANDNLLATINFWVYSIVIKIIPCIALTILSLRLISALIDTKRRREALTSGSRKTPRIAEKERQTDRTTRMLLAVLLLFLITEFPQGVLVLLSIYRGQDFFTDCYQKLGEIMDILALINSAINFMIYCAMSRQFRTTFSLLFRPRWLPVPQVENAQNNHTTTMVTQV*

>Picle_NPFR1 / Full-length

MACSEVTPSNVENCIKMELNGTFNFSLNDAIEILLEHTREDKNVDPVTEAVLIVVYGCLMAAGILANFIVIFVVARRPSMQTARNLYIANLTVSDLFLCLVCMPFTLIAILRRRWILGLVLCKLVPAVQGTNIMVSIGTITVIALDRYWTIVRGQDHARSRNRVIISIILVWVFSIIAVLPVICYQVVEEIKFNQLVLYETCIEKWSSQRLKVGYAACVLMVQAVIPALVVGLVHAQIAAYLNAHARTQRDSRRAQRELQRNRRTTLLLSGVAVLFAVSWLPLSVFSLLADLCLVSTDSLYVTLAACHVMAMTSAVSNPVVYGWLNSNFRRELVQVLPRWCRRPAVESSQEPSPTLLLCQNGQKPQHNNPATTYTAL*

>Picle_NPFR2 / Full-length

MFGGGLEDGGSLLLPADASGLMAAVAGMSPQNTSSEPLFNFSMKEALEILQEHQRNEKVLVPTTEIVIIIVYSILMTAGVFCNALVCFVVARQCARKHHQAGPSPRNMYIVNLALADLLLCLVCMPFTLVSILKRRWTLGLLLCKMVPAIQGANIMVSAGTISAIALDRYFTIVQTPRGPVCRTARCSVAATIATIWSLSFILMVPLLMYQEVDIVTAGDLVLYEACIERWPSRTFQASYTIVISVAQFLLPVLVLSIIHAKISSYLSLHLSSPPVDPSCKRAKREWRRNRRTMMILSCIAVVFALSWLPMTAFTIIFEFHPFLIKPTSTLYLVFALCHVTAMSTAVTNPLMYGWLNTNFRREFRVLGSTGVEKAREARRRLSTAHSVALRHDRRTSVTCFTTMTSSNTRPSTSVTLLPQAAMETI*

>Picle_OrphanR1 / Full-length

MVPGPQEAGAIVSGQVGEIPHKWLEVIVHFLMGFRNDTVDFSRPHLRPSVATVYPLFVFLYALLVTAGTVANLAMIASIVKDKLYRDQTYCYLINLGLANIVECLLVLPVSLMILLVQNWVFGSFLCYFLPMLQDIPLHVSTLTVLLIAWDRLRFLEDPMKPRIPAFVCATGSWLMAICIVLPYPIYTTYLDLGKYIGQFQGVGICAINLQDDMQEYMRGLFIAMYVLPLAVTAYLYIRMSRKLQAEEGPLPVMMFEGVRPRVSAGSHDFRGEDGQFRPHVPNRHETYEVELDMRKEKRTHKYLGTIATVFGICLCPLMVLRVAKLALTETYENSGHFDITYTMFVWLAFLPTTTTPALYASWRMSRSTKERLRGYLRLSSRRRRPEAAVGYTARESGNS*

>Picle_OrphanR3 / Partial

VTIFSLTAALSLTGNITVILVLSLGHRSSSELKSFLINLAVSDVAMAIFSIPFTYTMFLLGRWIFEPWFCPVVMTMQHTSVLVSVYTLTAIGIDRYKAITNPLGRRATASRNGLVISMIWLFSFFISSFQFRISWATSFFYDKQYYYKCQEHWDT

>Picle_OrphanR4 / Partial

CDLDLPPPDYFSHRYRVIGTVFQGTIFVIGVLGNAFVVAVVVRVRSLRSPTNCYLLSLAIADTIVLLASVPNEIFSYYMVGNRWVWGEAGCRVIIFFQNLGINSSSLSLIAFTVERYIAICHPMKAHKMCTIGRAKRITYGVWVFACVY

>Picle_EleR / Partial

DRSRYKRTVTDRFGTRGGSDSLVLQVIKMLVAVVVVFVVCWGPMLTDNLLTAWSVLPTTRTNQLKHMATAFHLMAYFNSCVNPIVYGFMSKNFRDSFSKALCCRGRAPRRQLSVSHTRTTSLRTQDTRAYIN*

>Picle_OrphanR7 / Partial

…NSSYRLGFDSRYTQEWTFFFTFYSQFGPRTFEARLEATILLCVLFFSVLTNSFLIAALLKARNKTVTNCFLMSLCFADSLFALGIPAVVAARLNPHWVIGDFLCRLLPYSQLTCGFTVLWSLTFISVERYRCLTLDPRLKLSVSSAGMVVLFMWLVAMLLFSPILFWFRHEEDLNICTIMFPRAPPVKVSLLFTVVVTIFVCLLPMAILVFNYQRIFMKMMETRQRWSTPCTLSSSLTRQEEQRMQKHIRVMRLSLMNVAVVLLMWLPITVVLCLIYVDGDRSIEDTGFFLRSHHFLAALSIALLNTAINPVLTGAIKFACCRSDAQGSSSFSRLIRIGSSFN*

>Picle_OrphanR8 / Partial

…FKCNNHFCVPLDLVCNFEDDCGDNSDEMKCLHRKCWNPEFRCSNGECLRPAYLCDGRADCKDGSDETNCGPEYFRKCGDGTLKHKYYWCDGWPDCPDNHADELNCKECDGKEDFLCPNGRCIKKANVCDAQCDCASDNGTYCEDEIGCSNYYTQVEGINLCVVGSTLSCTVPGHTRDNSRCIAGKYLCDQVVHCHSFYSDEYGCVYDEAEKAESNVRTFRCLDNRTLPEMLLCDHKYDCLHGDDEEGCQVPSSCGENMFRCKNGQCLEDSARCNVTIECWDKSDEIGCLDVVCQEGMKKCIQGGQCIPESSWCNFFIDCPDASDEKNCLTTQCKEDEFTCDNGQCIDKKHQCFNSGKSYEGCADSSHLQNCKDWLCEPWQFRCHLGPCLDPSLLCNGR…

>Picle_PKR1 / Full-length

MEELESYEFLNDTITDWPKRDPLYIVIPMTVLYSLIFVTGLIGNVSTCVVIGRNRHMHTATNYYLFSLAISDLLLLVSGLPQEMYYIWSRYPYVFGEAFCLLRGLAAETSANATVLTITAFTVERYVAICHPFLSHTMSKLSRAFRLILVIWVVALAFAIPQALQFGVVEEEDNPDSAQCAVKRILIEHSFELSTLLFFVGPMTLITILYALIGLNLRRSAIISKSGGSFMADKRRLPARNSSSQRVLKMLVAVVVAFFICWAPFHAQRLVAIYGSSGHNTPSSPFMLSLYSVVTYASGILYYFSTTVNPILYHIMSLKFREAFKSTWCRKRKRRTYLILSRGLESGKSVTDSAGSPLPNSRQRKQQLPEPDISNSSLRDVDKMALEDELSAYMRYRQDLMVRQA*

>Picle_PKR2 / Full-length

MADSTEMANGDQPYILLLLTGLYSLIFVTGLIGNVSTCIVIAKNRHMHTATNYYLFSLAVSDLLLLVWGLPQDVYLLWAKSYVFGETFCILTGIASETSANATVLTITAFTMERYIAICHPFLTHTVSKLSRAVKFVIVIWVVALFLAVPQAIQFGVIRVPTRETPNGTVLEESLHCSLKSDRQWLQYSFQISTVVFFVAPMSLISGLYLLIGVKLRRSRLIKRPSSEPLRAQKHVIRMLVAVVVGFFICWAPFHAQRLLVIYSPTVDRSSLLVTVYTALTHISGVLYYLSTTINPLLYNIMSLKFREAFKDSLAQHCGRRAKGLENHGRTYSVLSRGNTQVVRAIDSRRLKQTAPAPATISNSSLQDAGVEYSGAELASYMGQLNNSR*

>Picle_ProR / Full-length

MNGTAENTLGTSKSKSFYFLQQCRFWIPNVVAPIVFIIGLFGNVMTIIVLTRKRMSGSTNSYLTALAVSDLMYLIFYMTLCFEHHLNDSKYILYWKYWRFALWFADATAAISTWLTVSFTLERYIAVCHPLKGKVVCTEARARKVIGFVVMFGLISTVTTPFEWNAQIHINPETNETSPIQYSSLGKNKTYKTIFYWFSGITFVFIPLILLGTLNYLLINAVRISQKKRKNLTEGNSRKNYKEKQENKITIALVAVVFLFLLCQIPSAITLILGIFYTPKANTDGYNIVHAFGNIFNLLLAVNAAFNFLLYCAMSDKYRQTMYRTFCPSLAMKHQRANTFSSNASYRSSTHR*

>Picle_SIFaR1 / Full-length

MDVFVEALLPGNDTSPEEPTLQEINATDFYRHGLSMSTAYTLAYALVFFIGLVGNYCVILVVYRSPRMRNVTNYFIVNLAVADMLVLVFCLPATLVNTIFVPWVMGWLMCKTVPYVQGVSVTASIYSLIAVSLDRFLAIWWPLKCQITTRRARIMIAIIWLIAGTVTLPWAIFFELVKYDENSDIMFCVEQWPEYFNGHLYFLFGNLLFCYILPMILITLCYVLIWVKVCHRHIPSDSKDAQMERMQQKSKVKVVKMLVVVVILFVLSWMPLYIIVAMYKFGINRSEWEDEMLNALLPIAQWLGAANSCINPVLYAFFNNKFRRGFAAIIKSKKCCGTLRYYETVVRANSTSTSLRKSSYYVTNNNNNSSRRQLSLDTNVSYISNNSGV*

>Picle_SIFaR2 / Full-length

MNLTLLNITTYNSTEVQEFRYSFPVTVFFCLAYTSVFIIGVIGNVLVVSVVLRTPRMRSPTNLFIANLALADLLVNFICLPFTLVGNVTISWKLGWLICKTIPYLQGVSVNASINTLVAISVERCLAICYPMKWRISTTVVKITVIVIWMISLSITFPWAVYFKYGEDSQMRTCTEEWPDRYSESVYFIVAHLFMCYILPLILISICYIMIWKRVVTRKLPGETHAHGEQLIQKSKMKVIKMLLVVIVLFACSWLPLYIICSRVKLGGEIGSMELAIIEFFLPIAQWLGTSNSCINPILYAFFNKKFRAGFKAIVSSRSCCSTLRYDYRTQFTISSRDIGNGTQKVPTKRMTISAGVPVKPKRPDPNLRTMSLRTPLPSNNNFQDFRTYCHDLNCNGTFV*

>Picle_SKR / Full-length

PANRKMGEHWWEVGKVLIPLYSVIFLLGVVGNCLVILVLLRNRGMRTVTNVFLLNLAVSDLIMGVLCMPFTFVGFILRDFVFGHVMCRLIPFMQASSVAVSGWTLVCLSVERYYAICHPLRSRAWQTLDHAYRLIAAIWFGSFLVMSPIPAFSELIPINNGHKKCRERWPTLDYEKGYTILLDMLLLVVPLLVLVTTYSMVARTLWRVIKKQPPQNNENSERKKITWNQSSRRGSPNLLRRSNTEKALKKKKRVVKMLFAVVLEFFICWTPLYVINTIALFAPAAVYERLGYKGISFLHLLAYCSSCCNPITYCFMNSSFRKAFLKVFGCLREEKMNS*

>Picle_sNPFR / Full-length

MNVSVGNQTGLDIIEDKAVQAVFCLLYTSIFVLGLFGNVLVCYVVGRNRAMHTVTNCFITNLALSDILLCTLAVPFTPLYSFLGRWIFGGALCHLVAYAQGTSVYISTLTLTSIAVDRFFVIIYPFKPRMKLTTCIFIIVCIWSFSLVMTIPYGLYMNHRPDQGLYYCEEKWPSENLRQVFGGMTATLQFVVPFFVISFCYIRVSVKLNDRARSKPGSKTSRKEEADRERKRRTNRMLIAMVAIFGVSWLPLNLINLINDIYIPTGNWRYYYLCFFVGHSIAMSSTCYNPFLYAWLNDNFRKEFKQVLPCFTASPPGPSGWRSERTCNGQETCQDTLLTTTAASVVVSPPHDDEKDAGGGADRQTGCGRLKSHDSVEVVLVAYNAGEDAVHIDNRRDKNQLLPL*

>Picle_NTLR / Partial

…GNAIVMWIVLAHRRMRTVTNYFLVNLSLSDLLMSLLNCIFNFIFMLDSHWPFGPFYCTVNNFVANVTVAASVFTLVAISVDRYMAIVRPLKHRMSRRKALLALLAIWMASGLLALPCLLYSTTRTRRYSNGQSSVFCMMQWPDGAYPTSTSEHVYNLVFLMVTYLGPVVAMAVCYSLMGRELWGSKSIGEQTQRQLDNIKSKRKVVRMFITVIAIFAICWLPYHGYFIYAYHNRSVTFSTYIPHLYLAFYWLAMSNAMVNPIIYYWMNNKFRVYFRQVICFCGCIKQHNIQNEPQSHQVNMEFIRRSKSCKRMGPLSEVSRLASDMSSCSSSTVHVEAVCLRFNQHPKRKYSKQYLESRYN*

>Picle_TKR1 / Partial

…GPGSDDQTKMSVDNDSLGWANGSGNFSLEEPENQFILPWWKQLIWTFLFAGMVIVATGGNLIVIWIVLAHKRMRTVTNYFLVNLAIADAMVSSLNVTFNYTYMVNSDWPFGRPYCKISQFVAVLSICASVFTLMAISVDRYMAIMHPLRPRMGRRMTLCIAAGIWVIGSAFSLPMLIFFTTYTQDFTNGDQRVICYAEWPDGSTTESYQEYLYNVLFMVMTYFIPIGSMGYTYVRVGIELWGSQSIGECTQRQLENIKSKRRVVKMMMVVVAIFAVCWLPFHIYFIITSHMPEIINLPYIQEVYLAIYWLAMSNSMYNPMIYFWMNTRFRRGFKQFFSWCPYVQVPPEGLTRREAVTTRFNYSCSGSPEANYRINRNGTLQRTSMANSSFETEKSICESYSYASFKRKRWSPNDPS*

>Picle_TKR2 / Full-length

MAVEVQVTWAVAFSFMLTAAIVGNSIVIWSVTAHKRMRTVTNYFLINLSMADLTMSIFNGIFNFVYMLNKNWSFGSFYCTINNYVANVTVAASVFTLTGITIDRYLAIIRPLQPRMTKSNAILGIAIIWSTSLLIGLPCLIFSTTLTHRSSGQTACIMKWPDGLPMTSIMDYLYNLLLFLFTYILPMVAMVCCYTAMGKELWGSRSIGELTQRQIDSIRSKRKVVKMFILIVIVFAVCWLPYHLYFIYTHHRKDVVYSRYVQHIYLGFYWLAMANAMMNPAIYYFMNPRFREYFRKAICEWDCCKRKQRSIKLERGETPPIRRFSHSYSRSGGTADEVSGSGGRKSGHHLIVSFKSQKNNNIANSHCQLIS*

>Picle_DH31R1 / Full-length

MMEDSFSRNDSDFLQCAIYINESKSKNFEGSYCEATWDGWSCWQETPAGTTAYAPCPKFITGFDPNLLAHKICTENGTWFRHPDSGMIWSNYTTCVNIEDLTLRQTVNNIYQTGYSISLVALLLSLFILSYFKSLRCPRNTLHMNLFTAFAANNFLWLLWYRLIVPFPEVVIENGVWCQCLHVILHYFLLSCYAWMLAEGLYLHTLLVSAFTSEQKLVRILTVFSWTAPLFFIVLYSVLRLAFDDTDQCWINDSDFSSVLVVLVVASMGLNLCFLCNIVRVVVGKLRAGPSQSSRPSQALLQALRATLLLLPLLGLNYLLTPFRPPNNHPLESYYEIISAFTASFQGLCVATLFCFFNGEVMAQIKRKWQYATFRTRANSYTATTVSCGRSTIVPTSEEENV*

>Picle_DH31R2 / Full-length

MIYTREEIDNITAQIKKECFAVAENYTEGLFCPREFDGWSCLNATPAGTVLHFPCPYFQLGFDPKRMAHRPCLENGTWFRHPETNKPWSNYTTCVDLEDYEMRNQVNFIYKAGYMISLAALLLSLFIFFYFRSLTCTRIQIHKNFFISLAINNLLWLIWYEAVLGNHTVINENGVGCQFLHVILQYFLVTTYFWGFCEGLYLYTLLVVTFLTESKVMVCLYLIGWGVPALIVSVYASLRISTNKDTDYCWIQESIYRWTLIIPVGLSMIANLIFLITIVRVVLTKLHAAQKTTPSNSFKDKNASIRSKRRSTVLSDTAFSERTKKAVRATLILIPLIGLQYVVMIVRPDQKTTWEYTYELTEAIVASSQGLCVALLFCFCNGEVTAAVRKKWRQCRLSKKRPWNSCSGVTSVSRHRRNLLDSDLLNQK*

>Picle_DH31R3 / Full-length

MGNYDNSSSASKANFEFFMKLKKDCEIRKRHQLEMFPAKVADRTGLRPYCSATFDGWSCWNTTPSGEIALAPCPNFVTGFDTKRFAFRKCMDNGSWFRHPDTGQPWSNYTTCVDMDDLEFRKIVNIIYVVGYSVSFAAIVISLIIFLTFRSLRCTRIAIHVQLFSSFAANDLMWIVWYKMVIGNPIVVQENRLMCQALHVLLQYLMVANYLWMFCEGLHLHLALVVVFVKDDNAMRWFYFIGWFLPAILAGIYALVRSSYPDETSQCWLNESHTQWILTVPVLLSMLASLGFLINVVRVLLTKLHCNSANPAPIGLRKAVRAALILVPLFGIHHILIPFRPEPNGPGERVYQVFSALLVSLQGFCVSLLFCFANVDVHAAFKAMARRIRRRATDNGNLTATQTREVV*

>Picle_DH44R1 / Full-length

MDDQELASNFSYHFEREECLQRWRSHTPEGWCPAVFDGALCWSPTGPGVLASQSCREEIHGVLYDTSKNATKFCHELGTWDNRTDYDQCQERTDILVTLTADDIEMTTVVYSIGYALSLIALSIALFIFIYFKEMRCLRNTIHTNLMFTYVLADFMWILSLTVSMHTDSVSCLILFTLLHYFILTNFFWMFVEGLYLYMLVVETFTRENIGLRAYLTIGWGLPVAVIMSWVLVRSNANDIPDSPPGTKQCTWMNQSWSDWIYQVPAILVLAVNLLFLVRIMWVLITKLRSANNAETEQYRKGCKALLVLIPLLGITYILFIAGPQSAVYSNIRALLLSTQGLSVGLLYCFLNTEVQNTLRHRWLRWREERSLATRAYAKDMSPNTRTESIRLYSRHEIVPYRKRESTGSESTTMTLVHTSRFSNGPRSPFLQPPSEPV*

>Picle_DH44R2 / Full-length

MASNLIATKNVTTEYLFELQKQQCSQLWSGPTPSGWCGAKFDGALCWGPTAPEHFAAQPCKEVINDVYYDTSKNATKFCDSNGDWNLTNYLDCQERDFMVLDAELTRNTELSTIIYSVGYTLSLITLSLALFIFIYCKEMRCLRNTIHTNLMFTYVLADFMWILSLTVQVSIHTDSISCLILFTLLHYFILTNFFWMFVEGLYLYMLVVETFTRENIGLRAYLAIGWGSPIPVIIVWIIARTSVQDPSTVSLMEVPDTMNQCTMMYTSMTDWIYIIPVLVVLVVNLIFLCMIMWVLITKLRSANNAETEQYRKGSKALLVLIPLLGITYILLIAGPDAEVYHSLRALLLSTQGLTVGLLYCFLNTEVQNTLRHRWQRWREERSLPHRTYTKDSSPNTRTESIR

>Picle_PDFR / Full-length

MSTDIDGDKREAELCQGRYTNLTDPGYCPAVWDKVLCWPPTQQGSLASQSCPSNQHGLDSSKFVWKKCLDSGRWEGEGESGWTNYTTCYGPEMLQLYRKLYSGSSPEMKISIAHRTRTLEIVGFSISLAALIVSLAIFSHFRVLRNNRTKIHKNLFVAMVAQVVVRLTLYIDQAIIRSKTTKTQGIDNTPILCEASYILLEYARTAMFMWMFIEGLYLHNVVTVRVFQETFRYRLYNCFGWGLPLVMTTAWAASIASKMKTKCWWGYNLTPYFWILEGPRFAVILLNFLFLLNIIRVLMVKLRESHTSEVERVRKAVRAAVVLLPLLGITNIVNMMEAPLDRQVWEFAVWSYTTHFLTSFQGLFVAVLYCFLNGEVRTAIRKSIYTYLSLRPHQFTPRRNSAYVSVGSTRPPEPQPETRV*

>Picle_PTHR / Full-length

MSAREIRQSHMETPLSKEEQNFILAQKRDECINGNSSFTPGYCPQVWDSILCWAEAPPGTLSSQNCPIYIAGFKSGNATKYCTENGTWFWFNNSVVNNTWTNFTQCINGPTGTIVTPPSSEGNLTLIPKYLPTVKAISQIGYSVSLCTLVIAFIILASFKKLRCPRNILHMHLFVSFILRAGLSLLKSTLFVRGLGLASDIAVAVDGQYIHDEMTLNWSCKLLISFWQYFILANYSWILMEGLYLHNLIFLALFSDTSAITTYIILGWGLPLLSVIPWVIVRATLDDRLCWTAYSNHNYFLLIRIPITVSILLNFGLFLNIVRVLLLKLTASISEEKRRFRRWAKSTLVLMPLFGVHYAIFLGMQYGFDEWVELVWLLCDTTFSSFQGFFVAVLYCFLNGEVQAEVLKRGRNQQQAYSMSEKSSVPILGIFRNKKNRKRGGRT

>Picle_EHR / Full-length

MHWLLTVVLLVCSSSAEELLLGYLTGSQRAPGDQEYTRPGLTISGAIAFAADQLSKGILKQKGHSLKFQVAETYGQEEASIRQTAALWTSNVSAYIGPQETCIHEARMAAAFNLPMISYYCTHYETSDKRQFPTFARTRPPDTQISKSVAATLLAFNWTQVTFFYLNSSESDMTPIAETILTTLEGAGVKVLDVATWSPVYHHGYMDNPFTAMVERTYMKTRIYLILGQYYEHLGLLVALEEKNLFDKGEYWVVGVDIDQYDEQQPDKYLQGLLQDEADARAVRGFRCYLGVTPSPSKGFENFTMVVNSYMEGPPFNYPNPVRSIGGVKTIRAEAAYLYDAVHLYVQAVVEALESGEDHRNGTAIIARLKRRHYHSAMGYMVYMDENGDAEGNYTLLVRRAGPSGKYGLYPVGVFTYRSGQSNPLPELHLTSDIEWVNGEVPIAEPPCGFTGEKCISHTMEIAGGVAGASLALLIVVSLVIYRNLKYEQELDSLLWKIDFKDILISEGTDDKDKTKVTHPLMRTSQVSLSSNPDCDFRYSSLFTTVGIYKGRVLAVKKVDKKSVDITRNMKKELKIMRDLRHDNLIPFIGACTDPPNICIVTEYCNRGSLKDILENEDVKLDNMFRASLVGDILQGLIFLHESPLKLHGNLKSSNCLVDSRWVVKLCDFGLLDFKSGSDFSSMWSLKHFCNELDSCYCSGLLYRAPELLRNTLSCNCIGTQKGDSYSFGIILYEIHTRRGPFGDTSGLSPRQILHRIIYPSSQPFRPPLELLESCLECVRECVQECWHEVPEDRPDIKAVRSRLRPMRKGMKPNIFDNMIAMMEKYANNLEALVDERTDQLVEEKKKTDALLYEMLPRCVAEQLKRGHKVEAESFDCVTIYFSDIVGFTAMSAESTPFQVVDFLNDLYTCFDSIIEAYDVYKVETIGDAYMVVSGLPVRNCDQHAAEIATMSLDLLNAVDRFKIRHRPNEKLKLRIGLHSGTVCAGVVGLKMPRYCLFGDTVNTASRMESNGLAQKIHCSEETKHLLDRLGGYHLEERGMIRMKGKGEVKTYFLIGEDPVWRERRRHGLQRGIRSSLKTKIPRAASFESPKRLRFAANHVESKRLEVITDSPIKKSSLEVNRCEHLSVSCPCIEEEIRPPGIISIDATSDSALLLHEPDTPLLTVTKVS*

>Picle_NPLPR / Full-length

MLRRLVSLTLAATICLAAVRKNLTVGYLTAVKGTLKDRQGLAISGAIAFALWEVNNNSTLLPNVTLDMKWVDTKGETVATARAVTDMICDGVYAFFGPEESCHVESIIAQARNIPMISYKCSDYRASAVPTFARTEPPNTQVTKSVVALLEYYDWHKFSIIIEETWAIVGDSLEKEALKKKMKVNHKISVMDRHLCCELKHNCCHTTYWYDVIQETKNSTRIYVFLGTAGTLIDMMSTMQALQMFENGEYIVIYVDTIPFYQREEAKYLWKPTMNNEEGQQNCKGGQMGLYKRAMSLLVVVSSPPSGDYEDFTRKVREYNSGEPFNFPTPQVFTNVSYVKFISIYAAYLYDSVKLYATALDDLLKGYPELNDTIIEEVASNGTKIIETIINRGSYKSITGTTIKLDSNGDSEGNFSVYSFIQNPVKENTSECGYHLRFVGQFQHQPNSTSPEFKAFARINWIHGTKPEDEPSCGFNNENCPKDDSQKGSIIAAIVLAIALFCATVSTISIYRKWKIEQEIEGLLWKIDVSELHDYHDIISSPSKISLVSATSFESRCGPQRFASTGQFRGLVVRVKEIKFSRKKDISRDIMKEMRLLREVRHDNINSFIGAVVEPMRILVVTDYCSKGSLYDIVENEDIKLDKMFIASLIHDLIKGMIFLHNSLLGVHGNLKSSNCVVTSRWVLQVSDFGLHEIRHCPECDSIGEHQYFRSLLWVAPEILRNVHNCRGTQKGDVYAFAIILHEVIGRRGPFGACGLDEPKEIVRMVKKIPEPDEVPFRPSISVVRDSEVGADFVIDLLNEAWSEDPEMRPDFPTIRARLKAMRDGKHRNIMDQMMEMMERYANNLEDLVNQRTLEVYEEKRKTEDLLHRMLPAPVAKRLTSGFGVEPESFDLVTIYFSDIVGFTAMSAESTPLQVVNFLNDLYTLFDSIIRGYDVYKVETIGDAYMVVSGLPLKNGDCHAGEIASMSLDLLRAVKNYQIAHRPGETLKLRIGIHTGPVVAGVVGLTMPRYCLFGDTVNTASRMESNGEPLRIHISESCKDALEKLSGYIIEPRGPVSMKGKGLVNTYWLVGATDEAIKPQEVDVNELPPPLFCRPRKSPRLLQTDSRRQSNDIRPLPQITEEPLLEPTALRGFKSLDPLPYQPKSKSLIKRSCHSLQEESGLGCGNGTLVNAPLLPDDKRWHSLETVPPSEPVTKKGLGGSSLRSWLFGIFNNNTYHHRSSDISLRKAGYQDLQPERESIV*

>Picle_OG2 / Full-length

MLWLLAVVGLASGARVRLSVIAPTSDHVQALPVILPIIELAVRNVSDRQHGLLPGVNITIAQRNSNCSSTYGPLAAMELYNHTDAFIGPVCDYVIAPVSRYSGVWGKPVITAGAQADHFFHKQEYPTLTRVMGSYRAVSIAMRHILNYFNWKKAGLLYFDYGATSSKGHSKCYLMLGPVFSSLGASSTHKNFDDSATVEQFKELLIYIAKSARIVVVCADPPVVREILLVAEELNMIDSGEYVFFNIELFNNQYNMHKPWYVKGDSPERNQRAKKAFTALLTVTARTPDSEAYRNFSDEIKVMAKERYNYTFGPDPVSTFVTAFYDAVLLYALALNETLSSGGNQSDGFALTKRMWNRKFEGITGEVNVDSNGDRITDYSLLDMDPESGEFRIVAHYIGTKEELVTINDTKIHWSGGRTSPPPDTPVCGFDNSLCKSMPIYAILSIALGSVVVVLFVASALMYRHYKLEAEIASMTWRVYPNEIVHVTPGKFRGSMYSLVRRGSQLTVFSEDGVSLFGDGRGQVFVPTAVYRGNKVAIKTINKNRIDLHRGFLLELKRMKDLHHDHLVRFYGACIDPPNCCLLTEYCPKGSLQDILENEQIKLDWMFRYSLMHDIVKGMAFLHHSDIRSHGSLKSSNCVVDSRFVLKIADFGVRTLRQYSSFVDDVDSYSYWKKLLWTAPELLRMQNPPLEGTQKGDVYSFAIIVHEILSRQGPWAMTEVSLTPREIVESVRSGGKCLRPSTAELCCDEEVSALMKRCWAEDPADRPDFNSLKTTIRKLNKDNESNNILDNLLSRMEQYANNLETLVDERTADYLEEKRKCEELLYQLLPKSVASQLILGQSVVAETYDSVTIYFSDIVGFTSLSAESTPLQVVELLNDLYTCFDSTIENFDVYKVETIGDAYMVVSGLPMRNGNLHAREIARMSLKLLQMVKSFTIRHRPWDQLKLRIGMHTGPCVAGVVGLKMPRYCLFGDTVNTSSRMESNGEALKIHVSPKTKEVLDTFGTFELELRGEIEMKGKGKMTTYWLLGEREPPPDKQEPSGNNTLPGSTISNTTIGQTVGCDTLEGSPISGNTLSDISVGNTITSPVLSRHQNNISKPTANHSSSITASTPLLQGDSG*

>Picle_OGC4 / Partial

…SKGAAPPEAINIMRQCWAEAPDMRPDFNTIHDLFKTLNHGRKANFVDTMFQMLEKYSNNLEDLIRERTEQLDMEKKKTEQLLNRMLPSYVADKLKLGMPVDPEEFSEVTIYFSDIVGFTTISAYSTPFEVVDLLNDLYTCFDATINAYNVYKVETIGDAYMVVGGL…

>Picle_OGC1 / Full-length

MFLYLLVCLAAVMSCGSQAPECPMPENESDGLLYQYPTERENNSLTIGFLGAYSQKPVFLGALPLAVKAVNSDERLLPGRRLGFLAANIGSSAVASPLPIRIMTQMRDQGTVAFIGPDGTCSAEALVAAAWNLPIISYKCADKLVSDKRVYHTFARTLPPSTKVSKSVVALLTRFKWFTFVIVYEKSPSFAQVKDAIKELATENGLIVTEEMEFKDDYIPKKIRELENIIDATFQRTRVYVFVGDHVALVDFVKCLRKRGLLEKGEYIVISADDEIYNPERRKNIIQRDYLDPWLQDPWFHHPGVRPVEYDIHGFRSVLKLTTSHPSNPEYKSICEEVKKISTRPPFCVPHHSTIFGSMPVPIHAAHLYDAVMIYARALTEVLRAGEDPRNGTIILQKILNRSYRSVLGYDVYIDNNGDAEFNLSVVAVLDDAESNGSARMSMQRVGYFSYPSNMTALPEFRYYNESRKIQWVGGLQPLAEPQCGFLGEKCKKDRWIIILIIGGIFCLLLVAGVFAFKHYRYEQKLACLLWKIDMREVTIIPTTTTSQKSNMIQVCRQSCLRSIVPDVSPKRAYTNIGFYKGNTVAIKTVYKRSVDLTRNIRKELKQVREVRHENLITFIGASVEAGNIKILMPYCTRGSLEDVLANDDLHLDNMFISSLIADIIKGMIYLHDSDIISHGNLRSSNCLVDSRWVLQISDFGLHEFKAGQEEPDESLEIRRRLWRAPELLRASHAHPRGTQKGDVYSFAIILYEVIGRQGPWGTTQLTPREITEQVKSGSGLRPDVKAFEISPSVIACMEDCWHEDPEQRPDFRFVRVKLKEMQAGLKPNIFDNMLAIMEKYASNLEGLVQERTNQLTEEKKKTDALLNRMLPKTVAEALKRGDPVEAESFECVTIYFSDIVGFTELSAVSTPLQVVELLNDLYTCCDSIISHYDVYKVETIGDAYMVVSGLPVRNGDRHAGEIASMALHMLTNIRRLEIRHRPGEPLKLRIGIHSGHCVAGVVGLKMPRYCLFGDTVNTASRMESTGEAFRIHISRTTQQLLERLGGYTCEERGNVYIKGKGEMQTYWLVGEDSERRMARLQDVVQSSISSERMSEAPDLLRRPTGNELLPDAGDATAARLLHQLHLRAVRAYSKHHSQEALPSGIPFHPHHNGHRSEPVITFKDTSTYV*

>Picle_OGC3 / Partial

…GLCTNRDYSLGHSPVLSCHANISYFQVVMFLNRVYKLFDALIEKYDVYKVETIGDSYMVASGLPVKNGKRHVSEIATMALDLLEGSTLFLIPHRPTERLRIRSGVHTGPVVAGIVGTKMPRYCLFGDTVNTASRMESTGEALKIHISLEMKKALDEVGGFKTEHRGFVDIKGKGVLDTYWLVSKEGGLKRNIDKELRECTDNHEPVYLRKFR*

>Picle_PTTHR / Full-length

MGIPNIFQILLLNLVVITGIHTLYFPAREVRLTVRVGSIPDILTKVQAFVNTGDKVSAVFSVSTNAVHPLPDSLLEVDIQGYVKWIDKPTEPKNLTAKIKATFQESEAETEIHVLVRPALKDPCNPPLEELCFAEGTIYQVSEDMKEPKALGEVGPVETPCNQQHPQYTVRPEKGKPARRPENAVASLAGGDGRTLRVNPRKIKGDTKLILECRKDKKLVTKPILLKLIPTTVHLPVVPSGSRRIVKSITKGSHLLLTWMVVKSGIKAEWVPPANKFPPHTIKLIKYEFRTGLVNATLLLMNLTWTEDVPKGEVNVTVAISVCANQNNASCTLRTWTVVSVKNSPAVTYPRGPIRISPSPMARVAQPANARNLSSLSFSSALPLLMESPYRVTERAGIVYLLHWDSPALLDIPGVLVEWSINKSGVGLAWLPVLLVHTNCSRGHRDMTLWDICSESVNDRICTSKCGMAATPRGNGYCQWRGDKLTNSTNLTRNYSTCVTELLYCPDRWCDPLEELNPRICSQDCTKEVVVGILDNGTGIASGTGVCTCDSVGKCHCGPNHKEMEMPITINAPKNQPRQSPKPFQRQSPEICDFQCFMVLGIIGGSVISVLGISVYIIAYRGCNFKNKGRESSREYSVHALLPIPGPEARTPVEPILSNQYEIDPKWEIPRGRLIVEECLGEGEFGRVLKATARDLPGSPGFTTVAVKTLKDNAGSSELDDLMSEYQLLKEVSHPNVIKLLGACTAPGGPIYIIIEFCSLGSLRAYLRRHRNINTNSEAINIFQKVNDISPRDILSFAWQIAKGMAYLTEIKLVHRDLAARNILLATGKVCKISDFGLTRDVYEDDTYLKKSRGRVPVKWMAPESLSDHIYTSKSDVWSFGIVLWELVTLGSSPYPGITVQNLYHLLKSGYRMQRPHNCSEQLYEIMNECWSSSPAERPSFAYLVDKFERMLEDGSDYLDCNIKMVSNPAYFASTEDQGITGNELDIRFTMDLRDTSELENRLKYENEMINKNTSYDIPKPISCIVMSDLLMKDEQR*

>Picle_InR1 / Full-length

MVREWKICDETLWKFLFGVFWILAVAIDTNAYLRTTDISTVRKEKICQSTDIRNSVSMFSQLQGCQVVEGFVQIVLIDNADELDYSNISFPELTEITDYLLLYRVNGLRSLGKIFPNLTVIRGNTLFENYALVVFELLHLQEIGLVSLTDIVRGAVLFAKNPTLCFVDTIDWDRIAYNAKGEHYISANKLQNECPMCPANNHSCATSLIDGGSLCWNSQHCQKVCPAHCNESCNSNGDCCHSSCLGGCHGPPNSDQCVACRYFNFQRRCVKKCPNNTYEYLNRRCILESECYGLSKPRETNKKINRRNNPWKPFKGQCVLECPTGYLETVDISNDQERYKCEACKGICKKECPGANVDSIAAAQELRGCTIIRGALEIQIRGGVNVVKELEDNLNTIEEIDDYLKIVRSFPIVSLNFLRNLRVIHGHKLESSRSALIVLDNQNLVELWDWKSRKNEFRIDQGRLFFHFNPKLCLSEIDKLKIAAGLGDYTEHEVASSSNGDKVACNIKELKVTVYKKNSIAALIRWNQFEHYDSRTLLGYVVYSIEAPYQNVTMYDGRDACGGDGWQVDDVSASENPVKDGEKQEINHILTRLKPYTQYAFYVKTYTVATEKSGAQSRIQYFRTEPDRPSEPRGLTAFSNSSSELVIHWQPPAHPNGNLTYYIVTGHWEKDNEEFIEQRNYCLEPISLPPDTHKITPKPKIEEHDELTDDKLNSCPCKRKLVDNKQREKEIQLEDHLQNQIYVKRYNNREKRDISSQVIEKSKSLLPNQKEETESHSFINGTQFTTSVDGNTSTLLMKNLRHFALYNIAVRACRKLEEGENSTKNNTCSEEAIVTARTMRLATADNIESKYFIWEISNKSLGVVQLKWNEPENPNSLIVTYEIEYKRSDIENYKPTVECITRKHFVGSGNTYYLRNLGPGNYSLRLRATSLSGNGYYTPYQYFVIEEEYTFTSHLLYIMIILFMLALSFLLVAAFFLRRQYSQLPNRKLIASVNPEYVPTVYEPDDWEVSRSRVELLGEIGQGSFGMVYEGRLYSPEGSSEQLCAVKTVNVQAADRERIEFLNEASVMKAFNTHHVVKLLGVVSQGQPTLVIMELMKRGDLKSYLRSHRPDSGNSNIVPPSLKRILRMAAEIADGMAYLAAKKFVHRDLAARNCMVAEDLTVKIGDFGMTRDIYETEYYRKGSKGLLPVRWMAPESLKDGIFTSYSDAWSYGVVLYEMATLASQPYQGLSNDQVLRYVIDGGIMERPENCPDKLYSIMRLCWEHKPSSRPSFIELVTLLQNDVGIDFHDVSFYHSEEGVEYRSQITSEDTPLRVSREIEDFSLSEDEDFKEPRSSTSSKISNGSTNANGYVPQQIVTTKC*

>Picle_InR2 / Full-length

MHAALGLYVLWLAYLGAARVCPSMDIRNSASALLNLEPCSRIQGSLQIVLMENETESSFANLSFPRLSEVSYYVIFYRVHGLTSIGKLFPNLAIIGGDVLSLDYALIITQMPHLKEIDLSELRLILRGSVAIFKNPKLCYVDTVDWDIIATTGSNFVTPAKDRSLCPGCGHCPPKGNCWSRNHCQVLQWGTDCHSECLGGCTGPSPEQCTACKNFIHGKKCVSSCPPLTYEFESKRCVTKNECLTMNNTLLRPGKTETKENWFVWDSLCVNSCPRGLERDIRLGCKKCIGRCIMYCKGANVESIQSAQQLRYCTHIEGSLVIQLRSGNQSFIQRELEDNLGHIEEIMGYLKVLRSFPLANLNFLKNLRVIHGSKLFRDPMNISFIMLENQNLQTIWDWDNRPAKRNFTILAGRLFFHYNPSLCLKHIYELGRIAGIEEITNSEVAKESNGNKFACNIIDLNIEIHFRNSTCITILIGPPKFRDSENQENYLLRYMAYYIKAPYRNVTADYETNECGSYRWTVDDVMNYPEKNSTKGVYHTLSHLEPDTQYAIFVKTYTIDSTGGQSTILYERTLPSKPTSPVDLHGASFSSDSVLLEWEPPERKNGHITEYVVTGFSRHEETKMLSELDFCLELFRDTLSNKKPEVTTILPEFKTQIGVEKHDCCADEEPIKLEQPEPICHNKNSKEAKVVSTFINSKEEASCEKYFYSLFENNIIKTGNPLNKRYVEYKKKFVNDLEEVKTKNHQLTIYEIVPGNRTNVTIKNLDHFMLYTFEVRACRKTDPAEEMITDKGRCSMSSTITLRTLRKQDADDVSWISTEVYNRSIQLKWEVPSKPNLMIFAFDLEYRRTDIPNFRPTIDCIKAQDYLTKGNYHLHGLDIGKYSIRVRAISLAGIGNFTEYKYISIYEFSSKNIILIVVGVAICVIIVAALAASVAYFWKKIRMRNEILIASVNPEYLGLPVVDEEWELPRERIQIIREMKRGNFGIVCEGILFPEEKRVAVKKVVETASDRDCVEFLNEATVMKRFTSAYHIVKLIGIVSRDWPQLVVMEMMDHGDLKTYLRECRNNKPPLPKEMLLMAAQIADGMAFMEATKHVHRDLAARNCMIREDLVVKIGDFGMARDIYETDYYRKGNKGLLPIRWMAPESLNDGIFSSKSDAWSYGIVLWEMATLASQPYQGMSNEQVLAHVVSGNKLDLPPIYPKPFKTLMLWCWKWKPKFRPSFLQILDELHEFSTRGFREVSFYDSAEGRDARQALNSELRQLELTANSASFHPASFQTASFQTVSFQTDNDT*

>Picle_Orphan_RTK / Full-length

MKVAAGLVGLFLVLCMVFPAEALTQCLRLPEVTPKRYVNYQGQTIDLTLGASDRVVHRLVTHVFQIFLSEVLGYPGVQIIRTNDSDVYDVFDNLSDNYDPNNLPKKYMVNLEVWLPPDLNTIHLLETKSVKECGNIAPPGRYSWFIPKNLSKPEYEDWRTFRQPEKAKLFSLTEIERNILKKYTKSPYNDTNQKYYCSEKYCKTGFYTPDKCRGRSCAVLVTTFPSETGFVVEDIERFNLLVEVVWVGPNLRTVINNLKSRNRIDGTSLVFLTHTPSEITLGSEHISVSFPPCDEYSNNVSCHYSSIRLVKLAWSRLVNAARPAYEALQKVELTTEDYKDLVEDYLSTGDMYRVACSWMKRHNDSYDSWRQWMPSDDERNELYIGGIFPLTTVETNSYTARGIVSSAIMAIEAVNANNLILRDYKLKMLLDDGQCQSDVVMKTFIDYILMNRYEKLVGILGPACSDTVEPLAGVSKHFHTVVISYSAEGSIFSREKYPYFFRTIGENKNYKYVYLKLLKDLGWERVASLTEDGTKYTEYISHTQDLLQANGIYFVANRKFPHDWEHDAMKQYLDELKHKNARIIIADVKDEAARSVMCEAYQRKMTAYEGWVWFLPHWLSPKWYEIPTNANKTIKCTTEEMKRAINGYLALTHQYYAADNETMQENITVGEWRIKYREKLNGSEVSNYAGYAYDAVWAYAYALDKLIKYNESYASSLHVENTTKKFAEMIHLTNFNGVSGHIMFSSGPSRISNVNVLQWIDEELHIIGSFFPNKNDKNNSEYASGRLSLNKSKIIWLNEDKSLPWDGRVAEDRCLLEGFANMIQSTCQTAIIVANVIGIFFLILIVFISFLVVRHKYEKKVKITQKYMKSWGIDLLSASTINSLDKWEISRDKIVINRKLGEGAFGFVYGGEAHLNEKGWVAVAVKTLKVGSSTEEKLDFLAEAEVMKRFEHKNIVQLLGVCTKNEPVYTIMEFMLYGDLKTFLLARRHLVNEKYPDETDEISCKKLTNMALDVARALSYLAELKYVHRDVASRNCLVNVNKVVKLGDFGMTRPMCESDYYRFNRKGMLPVRWMAPESLVLGVFTPASDVWSYGVLLYEIITFGSFPFQGLSNNQVLEFVKAGNTLSIPEGIKPQLECLIKSCWHKEYKKRPPASEIVEFLANNPRLLSPCLDVPLSSVQMEDTGEISITGTRGQRFSMGLRQRSSSHDIAEPLLPSPGRFRPMRRSSLGEEPQDSLL*

# Supplementary Figures and Tables

## Supplementary Tables

**Supplementary** **Table 1.** Primers used in this study

| **Primer name** | **Primer sequence** | **Amplicon Length（bp）** |
| --- | --- | --- |
| OKB-F | ATGTCCCCGATAGTATTCTGTCTAG | 730 |
| OKB-R | CTACATTTCTTACGAAGTTTGGTCC |  |
| CCAP-F | ATGTTGCTGTACATAACTGTACTCCTC | 195 |
| CCAP-R | CTATCTGCCTAACAAGTCTGTTGACG |  |

**Supplementary Table 2.** Information of the assembly and annotation of *P. lewisi* transcriptome.

| total transcripts | 209701813 |
| --- | --- |
| total unigenes | 74820301 |
| min length of unigenes | 301 |
| mean length of unigenes | 1203 |
| median length of unigenes | 641 |
| max length of unigenes | 28905 |
| N50 of unigenes | 2040 |
| N90 of unigenes | 466 |
| The precentage of annotated unigenes in NR (%) | 34.26 |
| The precentage of annotated unigenes in NT (%) | 22 |
| The precentage of annotated unigenes in KO (%) | 12.58 |
| The precentage of annotated unigenes in SwissProt (%) | 19.41 |
| The precentage of annotated unigenes in PFAM (%) | 22.9 |
| The precentage of annotated unigenes in GO (%) | 22.9 |
| The precentage of annotated unigenes in KOG (%) | 10.36 |
| The precentage of annotated unigenes in all Databases (%) | 5.77 |
| The precentage of annotated unigenes in at least one Database (%) | 41.88 |
| The precentage of annotated unigenes hit to Halyomorpha halys (%) | 64.06513985 |
| The precentage of annotated unigenes hit to Cryptotermes secundus (%) | 3.730993054 |
| The precentage of annotated unigenes hit to Cimex lectularius (%) | 2.792378449 |
| The precentage of annotated unigenes hit to Blattella germanica (%) | 1.619110193 |
| The precentage of annotated unigenes hit to Nilaparvata lugens (%) | 1.548714098 |
| The precentage of annotated unigenes hit to other species (%) | 26.24366435 |

**Supplementary Table 3.** FPKM values of neuropeptide transcripts from each sample repeat.

|  | **SG1** | **SG2** | **SG3** | **H1** | **H2** | **H3** | **G1** | **G2** | **G3** |
| --- | --- | --- | --- | --- | --- | --- | --- | --- | --- |
| ACP | 0 | 0 | 0 | 4.2 | 3.53 | 3.55 | 0 | 0 | 0 |
| ALP1 | 1.02 | 2.51 | 3.86 | 34.03 | 56.63 | 40.92 | 0.84 | 0.4 | 0.33 |
| ALP2 | 9.97 | 17.31 | 29.65 | 44.19 | 110.66 | 69.92 | 0 | 0 | 0 |
| ASTA | 0 | 0 | 0.09 | 16.38 | 25.94 | 24.94 | 9.06 | 6.8 | 3.55 |
| ASTB | 0.69 | 0.83 | 1.42 | 16.7 | 18.86 | 12.32 | 2.54 | 1.41 | 0.57 |
| ASTCC | 0.34 | 7.35 | 3.85 | 5.13 | 11.77 | 12.1 | 13.99 | 75.56 | 32.11 |
| ASTCCC | 2.97 | 3.59 | 3.37 | 22.61 | 29.97 | 24.71 | 4.71 | 3.16 | 2.56 |
| AT | 0 | 0 | 0.09 | 2.5 | 5.72 | 3.21 | 0.43 | 0.12 | 0.04 |
| AVLP | 0 | 0 | 0 | 14.72 | 37.25 | 35.39 | 0 | 0 | 0 |
| Burα | 0 | 0 | 0.48 | 10.49 | 20.18 | 12.68 | 0 | 0 | 0 |
| Burβ | 0 | 0 | 0.63 | 8.7 | 13.25 | 11.32 | 0 | 0 | 0 |
| CAPA | 0 | 0 | 0 | 3.5 | 13.24 | 6.4 | 0 | 0 | 0 |
| CCAP | 161.49 | 76.74 | 89.67 | 0.77 | 0.51 | 1.15 | 0.55 | 7.46 | 4.78 |
| CCHa1 | 0.1 | 0.1 | 0.08 | 20.06 | 24.38 | 14.74 | 0.1 | 0.02 | 0.05 |
| CCHa2 | 2.33 | 2.82 | 3.04 | 6.93 | 7.63 | 8.45 | 14.27 | 10.87 | 11.52 |
| CNMa | 0.34 | 0.33 | 0.41 | 11.88 | 9.84 | 9.27 | 13.54 | 19.92 | 19.83 |
| CNP | 0.02 | 0 | 0.17 | 6.39 | 8.81 | 7.2 | 0.24 | 0.22 | 0.44 |
| Crz | 0 | 0 | 0 | 2.67 | 2.71 | 1.93 | 0 | 0 | 0 |
| DH31 | 16.41 | 30.59 | 22.84 | 43.37 | 65.99 | 50.68 | 0.72 | 4.64 | 0.53 |
| DH44 | 0.34 | 0.13 | 0.41 | 18.1 | 25.34 | 17.3 | 1.27 | 1.32 | 2.08 |
| EH1 | 0 | 0 | 0 | 3.39 | 3.04 | 4.63 | 0 | 0 | 0 |
| EH2 | 0 | 0 | 0 | 8.53 | 11.1 | 7.15 | 0 | 0 | 0 |
| Ele | 0 | 0 | 0 | 1.55 | 3.21 | 3.07 | 0 | 0 | 0 |
| ETH | 8.85 | 9.09 | 12.64 | 62.27 | 57.09 | 58.07 | 16.17 | 13.27 | 5.87 |
| FMRFa | 0 | 0 | 0.12 | 4.79 | 7.43 | 4.34 | 0.22 | 0 | 0.31 |
| GPA2 | 0 | 0 | 0.55 | 4.33 | 7.52 | 4.84 | 0 | 0 | 0 |
| IDLSRF | 0 | 0.03 | 0.13 | 9.74 | 11.83 | 9.43 | 0.02 | 0.1 | 0.04 |
| ILP1 | 0.25 | 0.32 | 0.59 | 3.55 | 2.28 | 2.71 | 0.35 | 0.28 | 0.26 |
| ILP2 | 3.54 | 3.22 | 4.68 | 77.3 | 80.99 | 61.38 | 4.56 | 8.32 | 5 |
| ITG | 0.7 | 0.39 | 1.2 | 68.39 | 97.01 | 72.13 | 0.44 | 0.37 | 1.45 |
| ITP | 0.78 | 0.82 | 0.94 | 11.65 | 12.54 | 29.12 | 0.15 | 0.59 | 0.14 |
| LK | 0 | 0 | 0.16 | 8.29 | 10.44 | 6.84 | 0.04 | 0 | 0 |
| MS | 0.39 | 1.33 | 0.16 | 28.46 | 37.52 | 30.63 | 18.98 | 16.28 | 10.97 |
| NPA1 | 1.9 | 0 | 0.59 | 2.7 | 5.99 | 3.82 | 0.79 | 0.5 | 0 |
| NPA2 | 3.44 | 3.55 | 1.36 | 12.26 | 26.71 | 12.08 | 5.61 | 2.11 | 2.83 |
| NPA3 | 0 | 0 | 0 | 73.43 | 100.49 | 70.11 | 0 | 0 | 0 |
| NPA4 | 0 | 0 | 0.23 | 49.13 | 55.76 | 41.36 | 0 | 0 | 0 |
| NPA5 | 0 | 0 | 0.33 | 42.21 | 50.62 | 33.23 | 0 | 0 | 0 |
| NPA6 | 0 | 0 | 0 | 50.6 | 45.52 | 44.75 | 0 | 0 | 0 |
| NPA7 | 0 | 0 | 0 | 10.11 | 14.13 | 17.12 | 0 | 0 | 0 |
| NPF | 0.47 | 0.18 | 0 | 24.88 | 32.38 | 23.69 | 3.85 | 1.32 | 1.47 |
| NPLP | 0.04 | 0.02 | 1.57 | 217.41 | 287.26 | 234.22 | 0.11 | 0.06 | 0 |
| NTL | 0 | 0 | 0 | 7.34 | 16.87 | 14.67 | 0 | 0 | 0 |
| NVP | 0.67 | 0.55 | 3.3 | 92.15 | 135.88 | 103.18 | 8.84 | 6.66 | 4.29 |
| OKA | 27.99 | 18.8 | 36.29 | 11.58 | 17.6 | 13.55 | 0.4 | 2.44 | 0.39 |
| OKB | 3406.58 | 3288.63 | 2989.11 | 18.51 | 19.1 | 13.19 | 29.23 | 302.76 | 86.29 |
| PaOGS36577 | 0.12 | 0.72 | 1.52 | 61.94 | 76.79 | 62.43 | 4.24 | 3.17 | 2.88 |
| PDF | 0 | 0 | 0.21 | 10.44 | 13.36 | 13.31 | 0 | 0 | 0 |
| PK | 0.27 | 0 | 0 | 16.78 | 16.94 | 13.11 | 0 | 0 | 0 |
| Pro | 0.12 | 0 | 4.25 | 54.58 | 144.12 | 92.54 | 0.26 | 0.12 | 0.24 |
| PTH | 0.99 | 7.07 | 0 | 7.15 | 7.6 | 8.59 | 0.8 | 9.26 | 5.98 |
| PTTH | 0 | 0 | 0 | 1.26 | 2.47 | 3.6 | 0 | 0 | 0 |
| RFLa | 0 | 0.22 | 0.14 | 2.88 | 3.09 | 2.42 | 0.17 | 0 | 0 |
| RYa | 0.15 | 0 | 0.76 | 5.78 | 7.14 | 4.79 | 0 | 0.16 | 0 |
| SIFa | 0 | 0 | 0 | 31.3 | 61.96 | 43.53 | 0 | 0 | 0 |
| sNPF | 0.18 | 0.41 | 0.66 | 33.85 | 58.45 | 37.51 | 1.61 | 0.48 | 0.56 |
| TK | 1.8 | 1.95 | 8.12 | 34.33 | 43.31 | 31.26 | 8.14 | 7 | 9.41 |
|  |  | FPKM>10 | FPKM>1 |  |  |  |  |  |  |

**Supplementary Table 4.** FPKM values of neuropeptide receptor transcripts from each sample repeat.

| **Gene name** | **Class** | **SG1** | **SG2** | **SG3** | **H1** | **H2** | **H3** | **G1** | **G2** | **G3** |
| --- | --- | --- | --- | --- | --- | --- | --- | --- | --- | --- |
| Picle_EHR | RGC | 11.56 | 15.06 | 13.35 | 2.05 | 1.22 | 1.85 | 0.49 | 3.15 | 0.44 |
| Picle_NPLPR | RGC | 9.19 | 10.1 | 12.35 | 4 | 4.02 | 4.33 | 10.32 | 8.32 | 10.56 |
| Picle_OGC2 | RGC | 2.19 | 6.5 | 7.9 | 5.56 | 4.45 | 3.4 | 2.09 | 1.75 | 3.98 |
| Picle_OrphanR5 | GPCR_A | 3.88 | 4.63 | 6.01 | 1.63 | 1.01 | 0.75 | 0.22 | 0.39 | 0.33 |
| Picle_InR1 | RTK | 3.81 | 4.77 | 5.52 | 5.03 | 4.99 | 5.11 | 2.84 | 2.45 | 2.14 |
| Picle_CAPAR | GPCR_A | 0.51 | 5.07 | 5.29 | 0.7 | 2.09 | 0.34 | 10.97 | 8.13 | 11.14 |
| Picle_DH44R2 | GPCR_B | 2.12 | 2.51 | 2.37 | 1.98 | 1.32 | 1.37 | 0.32 | 0.07 | 0.39 |
| Picle_Orphan_RTK | RTK | 1.52 | 1.96 | 2.69 | 1.59 | 1.39 | 1.22 | 2.8 | 1.84 | 1.71 |
| Picle_InR2 | RTK | 1.63 | 1.55 | 1.32 | 1.1 | 1 | 0.69 | 0.67 | 0.62 | 0.38 |
| Picle_PTTHR | RTK | 1.41 | 1.27 | 1.5 | 4.05 | 3.86 | 3.47 | 2.75 | 1.89 | 0.75 |
| Picle_DH31R1 | GPCR_B | 0.71 | 1.15 | 2.16 | 4.57 | 3.51 | 2.6 | 2.27 | 1.28 | 0.2 |
| Picle_CCAPR2 | GPCR_A | 0.59 | 1.68 | 1.29 | 7.14 | 10.15 | 6.14 | 0.58 | 1.09 | 0.47 |
| Picle_TKR1 | GPCR_A | 0.31 | 1.27 | 1.6 | 0.65 | 0.26 | 0.57 | 0.03 | 0.06 | 0.64 |
| Picle_PKR2 | GPCR_A | 0.47 | 1.28 | 1.33 | 2.58 | 1.69 | 2 | 0.49 | 0.58 | 0.74 |
| Picle_CCHaR2 | GPCR_A | 0.06 | 1.01 | 1.16 | 0.64 | 0.42 | 0.84 | 0.04 | 0.03 | 0.1 |
| Picle_sNPFR | GPCR_A | 0.52 | 0.47 | 0.97 | 2.08 | 2.56 | 1.84 | 0.83 | 0.55 | 0.63 |
| Picle_CCHaR1 | GPCR_A | 0.4 | 0.38 | 0.95 | 0.21 | 0.27 | 0.3 | 0.13 | 0.06 | 0 |
| Picle_OrphanR7 | GPCR_A | 0.59 | 0.46 | 0.49 | 3.05 | 3.94 | 2.65 | 0.07 | 0.12 | 0 |
| Picle_CrzR | GPCR_A | 0.07 | 0.57 | 0.81 | 3.63 | 2.23 | 4 | 0.03 | 0.3 | 0.24 |
| Picle_GPAR1 | GPCR_A | 0.02 | 0.5 | 0.91 | 0.25 | 1.41 | 0.57 | 12.44 | 6.81 | 6.48 |
| Picle_LKR1 | GPCR_A | 0.28 | 0.58 | 0.57 | 0.79 | 0.7 | 1.33 | 2.14 | 0.08 | 0.08 |
| Picle_ProR | GPCR_A | 0.2 | 0.97 | 0.24 | 14.54 | 13.67 | 8.56 | 0.88 | 2.3 | 0 |
| Picle_CNMaR | GPCR_A | 0.23 | 0.68 | 0.4 | 0.38 | 1.16 | 0.93 | 0.23 | 0.08 | 0.19 |
| Picle_PDFR | GPCR_B | 0.51 | 0.45 | 0.33 | 6.91 | 7.52 | 4.69 | 0.04 | 0 | 0 |
| Picle_OGC3 | RGC | 0.39 | 0.43 | 0.47 | 5.65 | 5.76 | 7.21 | 0.52 | 0.81 | 0.08 |
| Picle_MSR | GPCR_A | 0.22 | 0.41 | 0.5 | 1.83 | 2.47 | 1.83 | 1.93 | 1.15 | 2.34 |
| Picle_AstCR | GPCR_A | 0.09 | 0.36 | 0.66 | 11.53 | 15.5 | 9.6 | 0.42 | 0.55 | 0.33 |
| Picle_GPAR2 | GPCR_A | 0.21 | 0.38 | 0.39 | 0.13 | 0.25 | 0.21 | 1.44 | 0.61 | 1.07 |
| Picle_AstBR | GPCR_A | 0.24 | 0.38 | 0.25 | 2.94 | 3.55 | 2.86 | 0.6 | 0.62 | 0.14 |
| Picle_PTHR | GPCR_B | 0.18 | 0.45 | 0.17 | 3.2 | 3.5 | 2.83 | 1.04 | 0.53 | 0.53 |
| Picle_BurR | GPCR_A | 0.19 | 0.28 | 0.28 | 2.43 | 2.86 | 2.16 | 0.14 | 0.15 | 0.06 |
| Picle_PKR1 | GPCR_A | 0 | 0.51 | 0.16 | 1.18 | 0.76 | 1.17 | 0 | 0.05 | 0.16 |
| Picle_ACPR | GPCR_A | 0.32 | 0.09 | 0.2 | 0 | 0.29 | 0 | 0 | 0 | 0 |
| Picle_NPFR1 | GPCR_A | 0.2 | 0.12 | 0.15 | 0.57 | 0.73 | 0.6 | 0.08 | 0.03 | 0.04 |
| Picle_OGC1 | RGC | 0.07 | 0.16 | 0.19 | 1.43 | 1.02 | 1.5 | 0.07 | 0.19 | 0.09 |
| Picle_ETHR | GPCR_A | 0.11 | 0.16 | 0.14 | 2.05 | 2.14 | 1.29 | 0.16 | 0.21 | 0.06 |
| Picle_OrphanR8 | GPCR_A | 0.28 | 0 | 0.13 | 0.07 | 0.33 | 0.65 | 0 | 0 | 0 |
| Picle_DH31R2 | GPCR_B | 0.16 | 0.09 | 0.1 | 1.09 | 0.84 | 0.78 | 0 | 0.21 | 0.21 |
| Picle_SKR | GPCR_A | 0 | 0.16 | 0.18 | 0.24 | 0.12 | 0.47 | 0.05 | 0 | 0 |
| Picle_CCAPR3 | GPCR_A | 0 | 0.26 | 0.05 | 1.96 | 3.35 | 2.96 | 0.26 | 0.25 | 0.12 |
| Picle_AstAR | GPCR_A | 0.16 | 0 | 0.13 | 0.46 | 0.49 | 0.45 | 0 | 0.11 | 0.11 |
| Picle_OrphanR1 | GPCR_A | 0 | 0.14 | 0.15 | 2.11 | 2.14 | 1.48 | 0 | 0 | 0 |
| Picle_CCAPR1 | GPCR_A | 0 | 0 | 0.24 | 2.36 | 4.34 | 2.86 | 0.04 | 0.11 | 0.11 |
| Picle_NPFR2 | GPCR_A | 0 | 0.16 | 0 | 3.35 | 4.44 | 3.59 | 0 | 0 | 0.04 |
| Picle_SIFaR2 | GPCR_A | 0.01 | 0.06 | 0.06 | 1.93 | 2.31 | 1.45 | 0 | 0.03 | 0.01 |
| Picle_DH31R3 | GPCR_B | 0.04 | 0.09 | 0 | 1.09 | 0.98 | 1.22 | 0 | 0 | 0 |
| Picle_ATR | GPCR_A | 0 | 0.1 | 0 | 0.58 | 0.33 | 0.1 | 0 | 0 | 0.1 |
| Picle_DH44R1 | GPCR_B | 0.03 | 0 | 0.07 | 2.4 | 3.2 | 2.63 | 0 | 0.03 | 0.03 |
| Picle_FMRFaR | GPCR_A | 0.02 | 0.03 | 0 | 0.09 | 0.05 | 0.3 | 0 | 0 | 0.02 |
| Picle_ILPR | GPCR_A | 0 | 0 | 0.05 | 0.39 | 0.37 | 0.24 | 0 | 0 | 0.09 |
| Picle_LKR2 | GPCR_A | 0 | 0 | 0 | 0.42 | 0.33 | 0.36 | 0 | 0 | 0 |
| Picle_OrphanR3 | GPCR_A | 0 | 0 | 0 | 0 | 1.78 | 0 | 0.26 | 0 | 0 |
| Picle_OrphanR4 | GPCR_A | 0 | 0 | 0 | 0.65 | 0 | 0 | 0 | 0 | 0 |
| Picle_SIFaR1 | GPCR_A | 0 | 0 | 0 | 0.37 | 0.5 | 0.37 | 0 | 0.04 | 0 |
| Picle_NTLR | GPCR_A | 0 | 0 | 0 | 0.2 | 0.32 | 0.24 | 0 | 0 | 0 |
| Picle_TKR2 | GPCR_A | 0 | 0 | 0 | 0.29 | 0.14 | 0.08 | 0 | 0 | 0 |
| Picle_OGC4 | RGC | 0 | 0 | 0 | 1.83 | 2.07 | 1.11 | 0 | 0 | 0 |
|  |  | FPKM>10 | FPKM>1 |  |  |  |  |  |  |  |

## Supplementary Figures

**
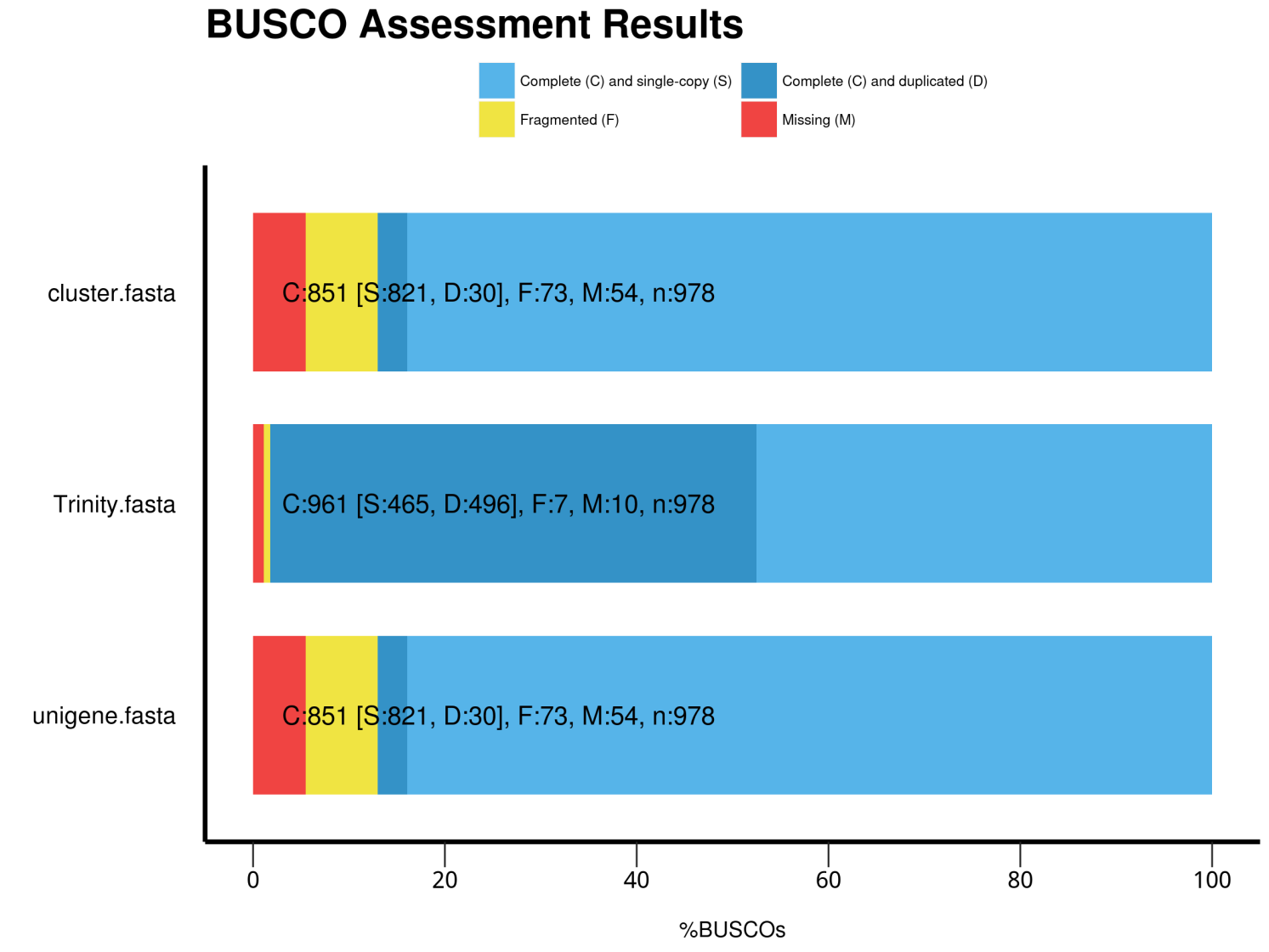
**

**Supplementary Figure 1.** The transcriptome completeness assessed with BUSCO.

**Supplementary Figure 2.** RT-PCR based amplification of two neuropeptide precursor transcripts from *P. lewisi*.
